# Dataset of numerical assessment on the combined effects of non-thermal plasma and water addition in hydrogen combustion

**DOI:** 10.1016/j.dib.2025.112405

**Published:** 2025-12-19

**Authors:** Ghazanfar Mehdi, Eljas Almusa, Mihiran Pathmika Galagedarage Don, Ossi Kaario, Zubair Ali shah, Muhammad Basit Chandio, Maria Grazia De Giorgi

**Affiliations:** a Department of Energy and Mechanical Engineering, Aalto University, Finland; b Faculty of Engineering and Applied Sciences, Memorial University of Newfoundland, Canada; c Department of Engineering Innovation, University of Salento, Italy

**Keywords:** Nanopulsed plasma discharge, Hydrogen combustion, Design of experiments, Water addition, NO emissions

## Abstract

This dataset contains raw and analyzed numerical simulation data, tables, and figures focused on the synergistic effects of nanosecond repetitively pulsed discharge (NRPD) plasma and water addition in hydrogen combustion. The data were generated using zero-dimensional (0D) plasma-assisted combustion simulations for H₂/H₂O/air mixtures. The simulation framework utilized a Design of Experiments (DoE) approach to systematically explore parameter interactions. This comprehensive numerical dataset offers insight into how plasma effects and water vapor influence hydrogen combustion and NO_x_ formation. The data are valuable for researchers and engineers seeking to design and optimize plasma-assisted hydrogen engines and low-emission combustion systems by providing information on ignition delay, radical formation, and emission behavior under various plasma–water conditions. It also supports the development and validation of chemical kinetic models and machine-learning-based combustion optimization frameworks using well-defined simulation parameters and responses.

Specifications Table

**Table:** 

Subject	Engineering & Materials science
Specific subject area	Mechanical Engineering.
Type of data	Raw, Analyzed, Table, and Figures.
Data collection	The data were obtained from zero-dimensional (0D) plasma-assisted combustion simulations using ChemPlasKin, a unified solver that integrates plasma and chemical kinetics through integrated modules (Cantera, CppBOLOS, muParser, and CVODES). Simulations were conducted for H₂/H₂O/air mixtures under varying inlet temperatures (300–900 K), equivalence ratios (0.4–1.2), pressures (0.5–1.5 atm), water contents (0–25 %), and plasma energies (5–9 mJ/cm³). A Design of Experiments (DoE) approach with Response Surface Methodology (RSM) in Design-Expert™ software was employed to explore parameter interactions and optimize ignition delay time, flame temperature, and NO_x_ emissions.
Data source location	Department of Energy and Mechanical Engineering, Aalto University, Espoo, Finland.
Data accessibility	Mehdi, Ghazanfar; Almusa, Eljas; Galagedarage Don, Mihiran Pathmika; Ossi, Kaario; Shah, Zubair Ali; Chandio, Muhammad Basit; De Giorgi, Maria Grazia (2025), “Dataset of Numerical assessment on the combined effects of non-thermal plasma and water addition in hydrogen combustion”, Mendeley Data, V1, doi: 10.17632/36h9hs69bm.1. https://data.mendeley.com/datasets/36h9hs69bm/1
Related research article	Mehdi, G., Galagedarage Don, M.P., Sehole, A.H., Kaario, O., Shah, Z.A., Chandio, M.B., De Giorgi, M.G., 2026. Numerical investigation of synergistic effects of nanopulsed plasma and water addition on hydrogen combustion and NO emissions. Int. J. Hydrogen Energy 198, 152,638. https://doi.org/10.1016/j.ijhydene.2025.152638.

## 1 Value of the Data

• This dataset offers a comprehensive numerical insight into the synergistic effects of nanosecond repetitively pulsed discharge (NRPD) plasma and water vapor addition on hydrogen combustion and NO_x_ formation.
• The data can help researchers and engineers design and optimize plasma-assisted hydrogen engines and low-emission combustion systems by understanding ignition delay, radical formation, and emission behavior under different plasma–water conditions.
• It supports the development and validation of chemical kinetic models and machine-learning-based combustion optimization frameworks using well-defined simulation parameters and responses.
• The dataset offers a valuable benchmark for comparing experimental or computational studies on plasma-assisted combustion, non-equilibrium plasma chemistry, and water–fuel interactions.
• The structured Design of Experiments (DoE) and Response Surface Methodology (RSM) data can be reused to explore sensitivity analyses, optimize operating ranges, or develop surrogate models for practical hydrogen combustion systems.

## 2 Background

This dataset provides the raw and analyzed numerical simulation outputs associated with the recently published full research article [1] published in the International Journal of Hydrogen Energy [1]. Recently, plasma-assisted combustion (PAC) has appeared as a promising strategy to boost fuel reactivity and improve overall combustion performance. Numerous studies have demonstrated that non-equilibrium plasma effectively promotes ignition and flame stabilization by accelerating fuel dissociation, enriching the radical pool, and locally increasing the mixture temperature [[2], [3], [4]].

The original motivation and context behind compiling this dataset were centered on investigating the synergistic effects of nanosecond repetitively pulsed discharge (NRPD) plasma and water addition in hydrogen combustion. The numerical study aimed to explore how these combined factors influence hydrogen combustion and formation. The theoretical and methodological background for generating data involves using zero-dimensional (0D) plasma-assisted combustion simulations for mixtures. The data were specifically generated using the ChemPlasKin solver, which is optimized for simulating chemical kinetics in non-equilibrium plasma environments and utilizes integrated modules like Cantera, CppBOLOS, muParser, and CVODES for detailed time-resolved predictions. Crucially, the simulation framework employed a Design of Experiments (DoE) approach with Response Surface Methodology (RSM) to systematically vary five key input parameters—temperature, equivalence ratio, pressure, water content, and plasma energy—to efficiently explore parameter interactions. The resulting matrix generated responses related to ignition delay time (IDT), flame temperature, and concentration.

This data adds significant value to the related research article [1] by providing the underlying information in a maximally accessible and citable format. The dataset, including the detailed raw and analyzed DoE test matrix (Table 1), figures, and profiles, offers comprehensive numerical insight into ignition delay, radical formation, and emission behavior under various plasma–water conditions. This material supports the development and validation of chemical kinetic models and machine-learning-based combustion optimization frameworks. Furthermore, the structure and data can be readily reused by researchers for sensitivity analyses, optimizing operating ranges, or developing surrogate models for practical hydrogen combustion systems.

**Table 1:** Design of Experiments test matrix considering five input parameters inlet temperature, equivalence ratio, water fraction, plasma energy and inlet pressure, which generates three responses, Ignition delay timing (IDT), Temperature and NO concentration.

Run	Inlet T (K)	ϕ	H_2_O (%)	Ei (mJ/cm3)	P (atm)	No of pulses	IDT (ms)	Temp (K)	●NO (ppm)
1	300	0.4	0	9	0.5	16	0.51	1422	1968.88
2	900	0.8	12.5	7	1	7	0.04	2469	2354
3	600	0.8	12.5	7	1	12	0.23	2301	2045.9
4	900	1.2	25	9	0.5	8	0.038	2508	3848.45
5	300	0.4	25	9	0.5	16	0.506	1377	2218.51
6	300	1.2	0	9	0.5	19	0.41	2343	3499.85
7	300	0.4	25	5	0.5	25	0.8	1377	1730.77
8	900	0.4	25	5	0.5	9	0.11	1884	2781.35
9	300	0.4	0	5	0.5	26	0.82	1422	1810.66
10	600	0.8	12.5	7	1	12	0.23	2301	2045.9
11	600	0.8	12.5	7	1	12	0.23	2301	2045.9
12	900	0.4	0	5	1.5	5	0.052	1930	636.296
13	600	0.8	12.5	7	1	12	0.23	2301	2045.9
14	300	0.4	25	5	1.5	43	1.43	1377	835.153
15	300	1.2	0	5	1.5	34	0.99	2367	949.191
16	600	0.8	12.5	7	1	12	0.23	2301	2045.9
17	300	0.8	12.5	7	1	25	0.72	2103	2060.82
18	300	0.4	0	9	1.5	29	0.96	1423	1104.3
19	300	0.4	25	9	1.5	28	0.92	1378	1104.84
20	900	1.2	25	9	1.5	5	0.043	2560	1088.02
21	900	1.2	25	5	0.5	12	0.075	2508	3054.74
22	300	1.2	0	9	1.5	23	0.66	2366	1212.55
23	600	0.4	12.5	7	1	10	0.27	1654	871.888
24	600	0.8	12.5	5	1	14	0.293	2300	1975.19
25	900	1.2	0	5	1.5	6	0.052	2679	1032.35
26	900	1.2	0	9	1.5	5	0.039	2678	1304.95
27	600	0.8	12.5	7	1	12	0.23	2301	2045.9
28	600	0.8	12.5	7	0.5	13	0.21	2280	4340.21
29	300	1.2	25	5	0.5	26	0.64	2188	2562.71
30	900	1.2	25	5	1.5	6	0.058	2560	821.602
31	600	0.8	0	7	1	12	0.25	2353	2417.01
32	900	0.4	25	9	1.5	3	0.039	1887	758.524
33	900	1.2	0	5	0.5	12	0.066	2611	3379.4
34	600	0.8	12.5	7	1.5	11	0.255	2308	1435.02
35	300	0.4	0	5	1.5	45	1.5	1422	848.789
36	300	1.2	25	5	1.5	33	0.98	2199	899.17
37	900	0.4	25	5	1.5	5	0.059	1888	559.636
38	300	1.2	25	9	0.5	18	0.4	2188	3095.17
39	600	0.8	25	7	1	11	0.22	2249	1988.1
40	300	1.2	0	5	0.5	28	0.66	2343	2914.35
41	600	0.8	12.5	9	1	10	0.19	2300	2438.52
42	600	1.2	12.5	7	1	12	0.22	2454	1618.46
43	900	1.2	0	9	0.5	9	0.042	2611	4215.54
44	300	1.2	25	9	1.5	22	0.65	2199	1134.53
45	900	0.4	0	5	0.5	9	0.09	1928	3265.15
46	900	0.4	0	9	0.5	7	0.075	1947	3834.78
47	600	0.8	12.5	7	1	12	0.23	2301	2045.9
48	900	0.4	25	9	0.5	7	0.077	1885	3387.62
49	600	0.8	12.5	7	1	12	0.23	2301	2045.9
50	900	0.4	0	9	1.5	4	0.038	1932	792.762

## 3 Data Description

Table 1 presents the Design of Experiments (DOE) test matrix, which systematically varies five key input parameters: inlet temperature, equivalence ratio, water fraction, plasma energy, and inlet pressure. This matrix enables comprehensive investigation of how these factors influence three primary combustion responses: ignition delay time (IDT), flame temperature, and ●NO concentration. The DOE approach facilitates efficient exploration of parameter interactions and sensitivities, supporting robust statistical modeling and reliable prediction of plasma-assisted combustion behavior under diverse operating conditions. This structured test matrix underpins the numerical simulation framework used in the study to analyze synergies between plasma effects and water vapor on hydrogen combustion and emissions.

### 3.1 Impact of different inlet pressures using fixed plasma energy and water concentration

**Fig. 1:**
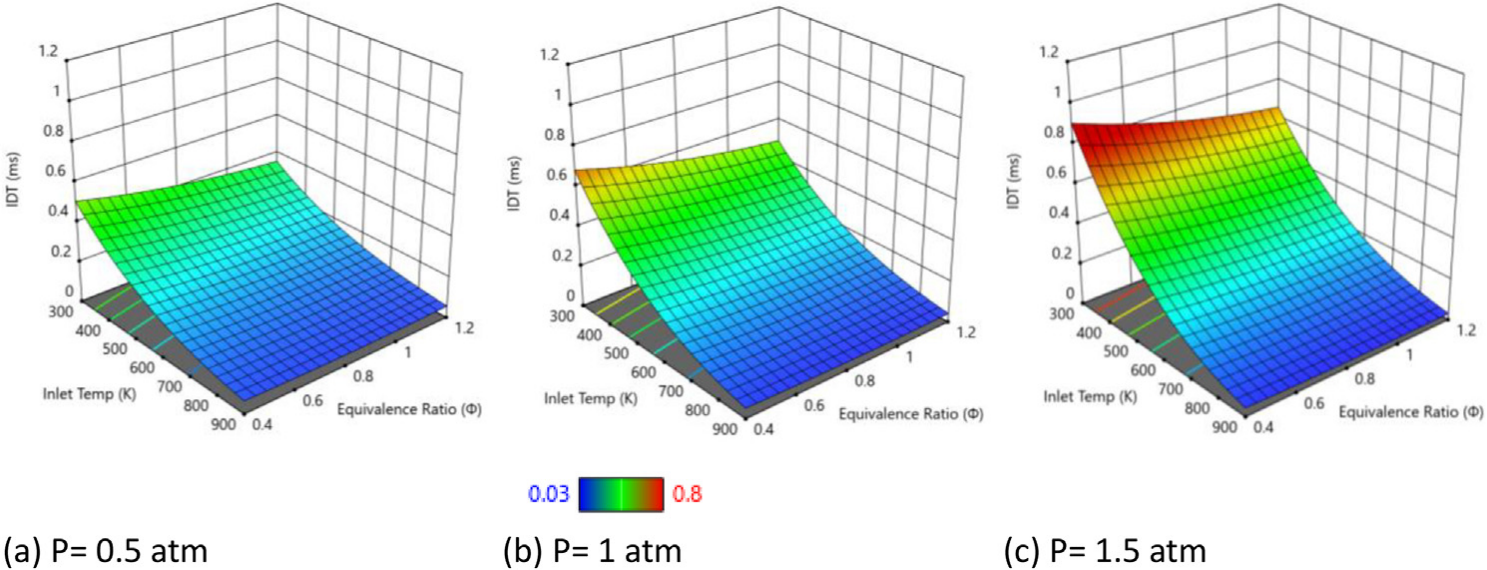
IDT variation with different inlet pressures at fixed at E_i_ = 9 mJ/cm^3^ and water concentration of 25 %.

Fig. 1 shows the influence of inlet pressure (0.5, 1, and 1.5 atm) on the ignition delay time (IDT) of a plasma-assisted hydrogen/air mixture with 25 % water vapor, under a constant NRPD plasma energy input of 9 mJ/cm³. Contrary to conventional thermal ignition behavior, the results indicate that IDT increases with increasing pressure, as evident from the shift toward red regions at higher pressures. At the lowest pressure (0.5 atm), IDT is the shortest across all equivalence ratios (φ = 0.2–1.2), with the most significant reduction observed near stoichiometric conditions (φ ≈ 1.0). As pressure increases to 1 atm, IDT rises moderately, and at 1.5 atm, the delay becomes the longest, particularly for lean mixtures (φ < 0.6). This trend persists despite the fixed plasma energy input, suggesting that pressure-induced kinetic effects dominate over plasma enhancement in this regime. This atypical trend may be attributed to the inhibitory effects of pressure on non-thermal plasma chemistry in the presence of water vapor. At low pressures (0.5 atm), the reduced gas density allows plasma-generated radicals (●O, ●H, ●OH) to persist longer, facilitating faster chain-branching reactions. As pressure increases, the higher collision frequency accelerates radical recombination (e.g., *H* + *O*₂ + *M* → HO₂ + *M*), forming less reactive hydroperoxyl (●HO₂) radicals. M denotes a generic “third body” molecule that participates only by taking up excess energy and stabilizing the product. This suppresses ignition kinetics, leading to longer IDT. Additionally, the high-water content can enhance third-body recombination effects, which become more prominent under elevated pressures, further prolonging the ignition delay. The fixed plasma energy (9 mJ/cm³) becomes less effective at high pressures due to rapid thermalization of deposited energy. The increased gas density enhances energy dissipation, reducing the availability of high-energy electrons for fuel dissociation. The 25 % water concentration acts as a third-body collider, further promoting radical recombination (e.g., *H* + OH + *H*₂O → H₂ + *H*₂O). At elevated pressures, water’s quenching effect is amplified, exacerbating the suppression of ignition. In conclusion, while higher pressure generally favors thermal ignition, under plasma-assisted and water-rich conditions, the increased pressure appears to retard the ignition process, leading to longer IDTs due to altered radical kinetics and enhanced quenching phenomena.

**Fig. 2:**
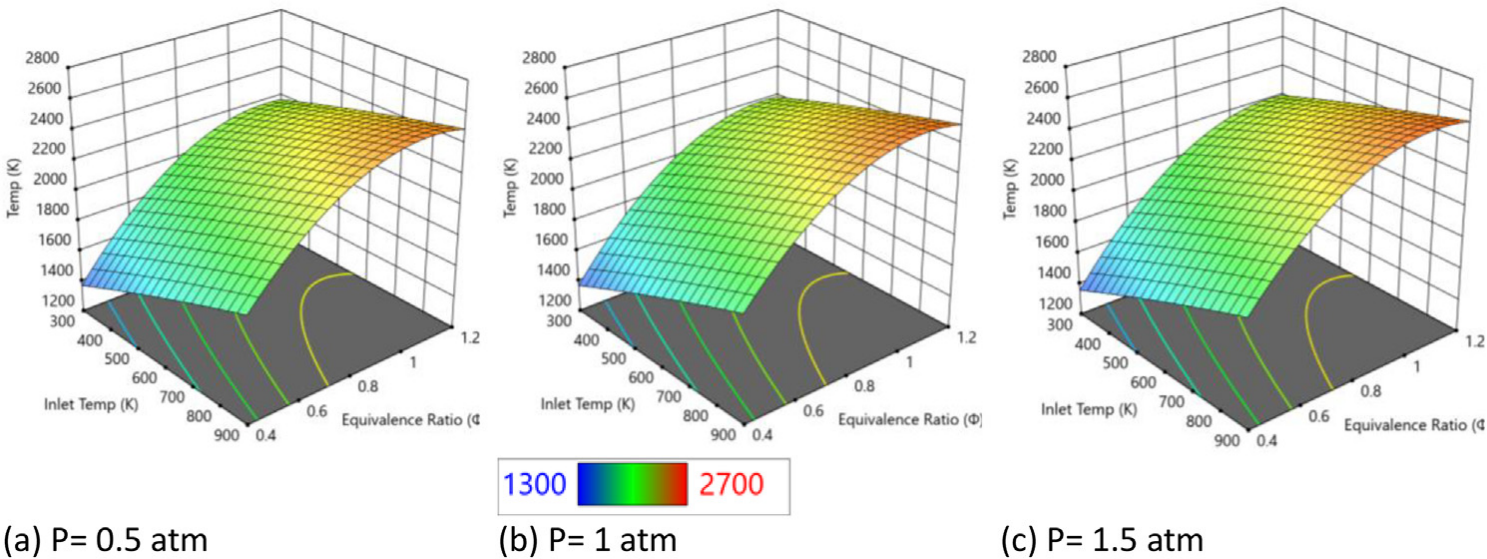
Temperature changes with different pressure inlets at fixed at E_i_ = 9 mJ/cm^3^ and water concentration of 25 %.

Fig. 2 shows temperature profiles under three different pressures (0.5 atm, 1 atm, and 1.5 atm) while maintaining a fixed nanosecond pulsed discharge energy (9 mJ/cm³) and water concentration (25 %). Each plot displays temperature ranges from approximately 1300 K (blue regions) to 2600 K (red-orange regions), with virtually identical spatial distributions and peak locations. The temperature surfaces keep consistent topological characteristics, with maximum temperatures occurring at high equivalence ratios (Φ > 1.0) and elevated inlet temperatures (>800 K), while minimum temperatures appear in lean mixture regions at lower inlet temperatures across all pressure conditions. The lowest temperature occurs at 0.5 atm due to inefficient plasma energy thermalization from sparse collisions. At 1 atm, temperatures rise slightly as improved collisional heating outweighs minor radical quenching. The highest (but still modest) temperatures are observed at 1.5 atm, where enhanced energy coupling to the gas dominates over recombination losses. Despite a 200 % pressure variation from 0.5 to 1.5 atm, the bulk gas temperatures show negligible differences. The temperature contours, peak values, and overall surface characteristics remain essentially unchanged across all three pressure conditions. This observation confirms the fundamental principle of non-thermal plasma operation, where plasma energy deposition occurs through electron-impact processes that are largely independent of bulk gas pressure within this range. The temperature independence across different pressures validates that the 9 mJ/cm³ plasma energy is preferentially directed into electronic excitation and chemical activation rather than translational heating of the bulk gas. The electron-impact cross sections and energy transfer mechanisms in nanosecond pulse discharges stay relatively constant within this pressure range, explaining why temperature distributions are unaffected by pressure variations. The 25 % water concentration appears to provide consistent thermal buffering effects across all pressure conditions. The consistent temperature distributions suggest that water vapor interactions with plasma species maintain their thermal characteristics regardless of pressure changes. The chemical pathways involving water dissociation and radical production appear to be governed by plasma energy rather than pressure-dependent thermal processes.

**Fig. 3:**
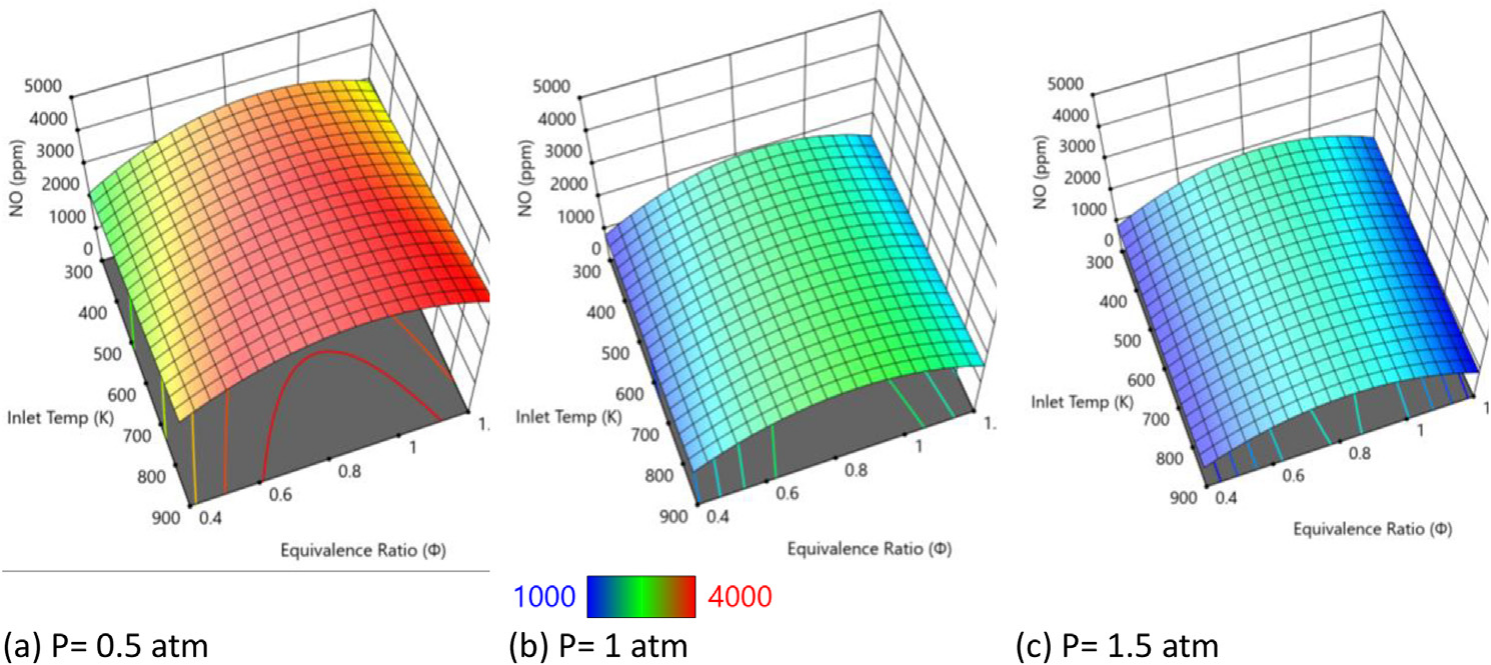
NO emissions with different pressure inlets at fixed at E_i_ = 9 mJ/cm^3^ and water concentration of 25 %.

Based on Fig. 3 showing NO emissions under varying pressure conditions (0.5–1.5 atm) with constant nanosecond plasma energy (9 mJ/cm³) and 25 % water concentration, a clear pressure-dependent trend appears that shows significant ●NO reduction with increasing pressure. The three 3D surface plots reveal a dramatic inverse relationship between pressure and ●NO emissions. At 0.5 atm (left plot), NO emissions are highest, with peak concentrations reaching approximately 4000+ ppm (red regions) across wide areas of the temperature-equivalence ratio space. At 1 atm (middle plot), NO emissions are substantially reduced, with peak values dropping to around 2000 ppm and a predominance of green coloring indicating lower concentrations. At 1.5 atm (right plot), NO emissions are minimized, with the surface dominated by blue coloring indicating the lowest NO levels throughout the operational range. The systematic ●NO reduction with increasing pressure reflects fundamental changes in plasma-assisted combustion chemistry. Higher pressures enhance three-body reaction pathways that are critical for ●NO consumption. Research on non-thermal plasma systems shows that increased pressure promotes radical recombination reactions and alters the balance between ●NO formation and destruction pathways [5]. The 25 % water concentration provides additional reactive species (●OH, ●H, ●O) through plasma-induced dissociation, and these species become more effective at consuming NO at higher pressures due to enhanced collision frequencies. At elevated pressures, three-body reactions become more significant due to increased collision frequencies. These reactions are particularly important for ●NO reduction pathways such as NO + *O* + *M* → NO₂ + *M* and subsequent conversion reactions. The higher molecular density at 1.5 atm compared to 0.5 atm increases the probability of these termolecular reactions, leading to more efficient NO conversion to other nitrogen species [6]. The constant plasma energy (9 mJ/cm³) continues to generate reactive radicals, but their effectiveness in ●NO reduction is amplified by pressure-enhanced reaction kinetics. The results demonstrate that pressure acts synergistically with non-thermal plasma to enhance NO reduction. While the plasma energy density remains constant at 9 mJ/cm³, the effectiveness of plasma-generated radicals in consuming ●NO increases with pressure. The electron-impact processes that generate reactive species from the 25 % water vapor are not strongly pressure-dependent in this range, but the subsequent chemical reactions involving these species show marked pressure sensitivity.

### 3.2 Impact of varying plasma energies using pure hydrogen at fixed atmospheric pressure

**Fig. 4:**
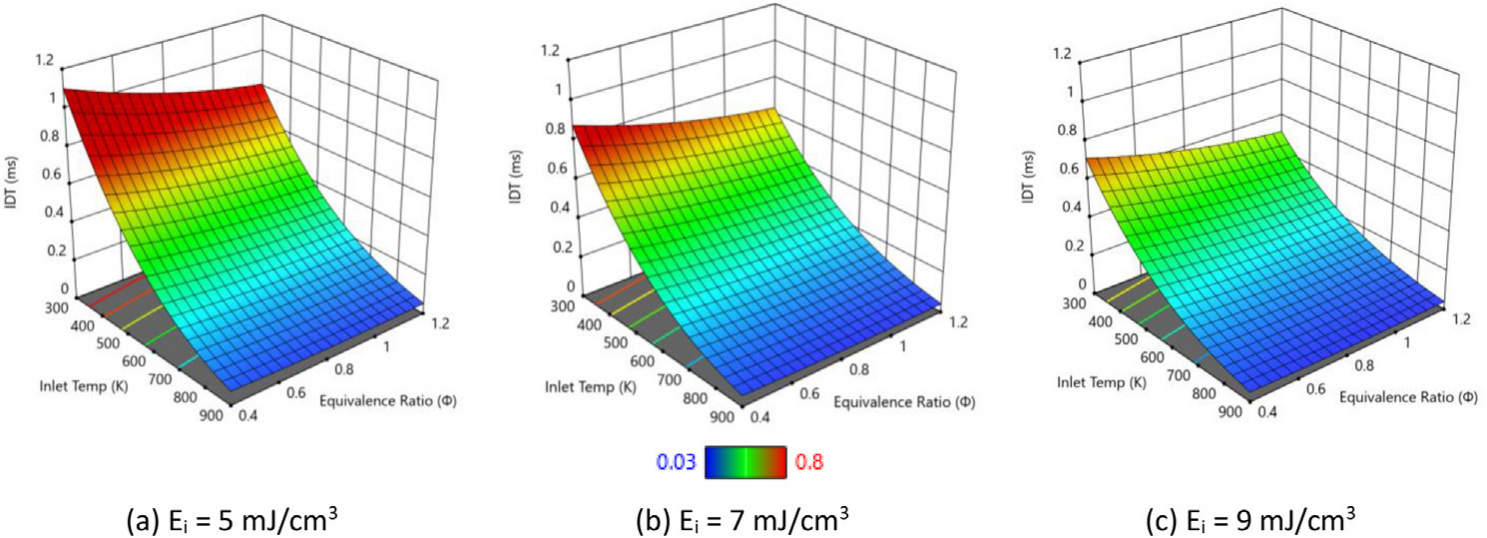
IDT variation with different pulse energies considering pure hydrogen and atmospheric pressure.

**Fig. 5:**
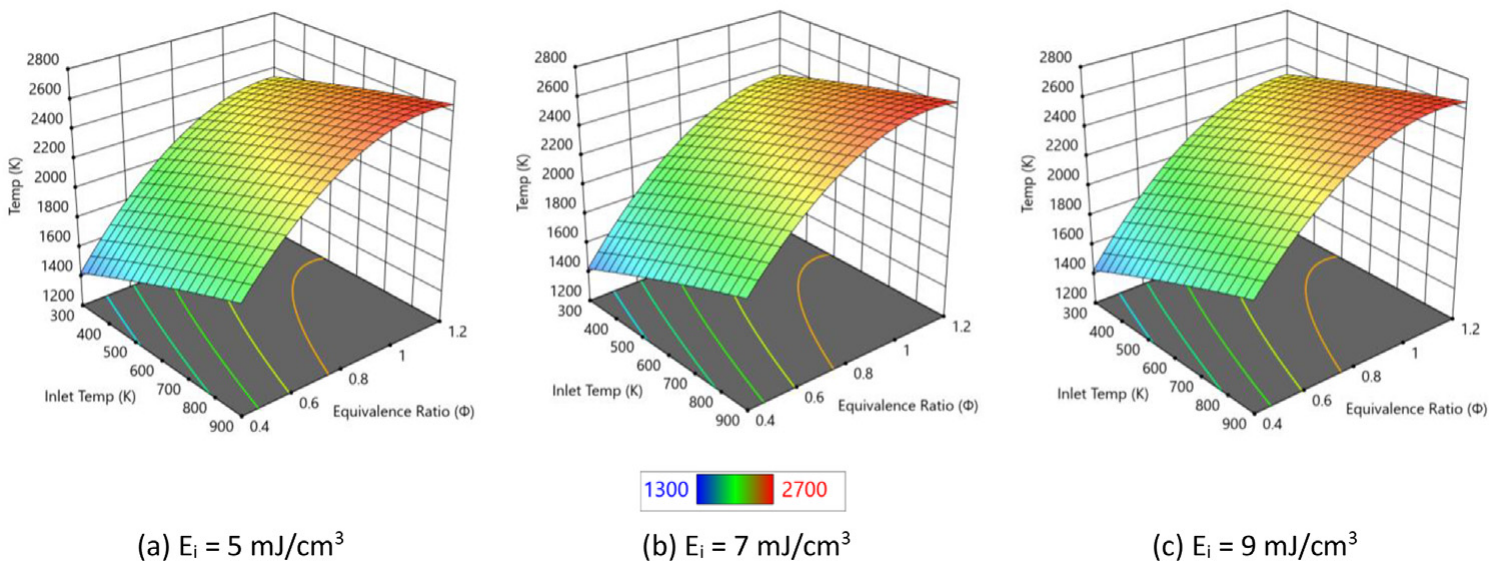
Influence of plasma energies on temperature of pure hydrogen.

In this section, we examine the impact of varying plasma energy inputs on IDT, peak combustion temperatures, and ●NO emissions in pure hydrogen combustion. Fig. 4 presents IDT trends for hydrogen/air mixtures under with varying plasma energies. Each 3D plot shows how IDT varies as a function of inlet temperature and equivalence ratio, with color indicating the magnitude of IDT (from higher values in red to lower values in blue). Across all three subfigures, IDT decreases with increasing inlet temperature and equivalence ratio, and comparison between panels (a), (b), and (c) illustrates how increasing the plasma energy input further shortens the ignition delay over the same range of conditions. Higher plasma pulse energy deposits more electrical energy per unit volume, which generates larger concentrations of excited species, ions, and radicals such as H, O, and OH during each pulse. These radicals directly participate in the chain-branching reactions $H_{2}+O\to H+\text{OH}$ and $H+O_{2}\to \text{OH}+O$, accelerating the overall reaction progress and shortening the ignition delay. Fig. 5 shows how the peak combustion temperature of pure hydrogen varies with inlet temperature and equivalence ratio for three plasma pulse energies (5, 7, and 9 mJ/cm³). Across all three subfigures, the peak temperature increases monotonically with inlet temperature and with equivalence ratio toward stoichiometric/rich conditions. The surfaces rise from lower temperatures (blue/green) to low inlet temperature and lean mixtures toward higher temperatures (yellow/red) at higher inlet temperature and near-stoichiometric equivalence ratios, indicating stronger heat release under these conditions. Comparing panels (a), (b), and (c), increasing the plasma energy slightly elevates the overall temperature levels but, more importantly, tends to steepen the temperature rise with equivalence ratio and inlet temperature.

**Fig. 6:**
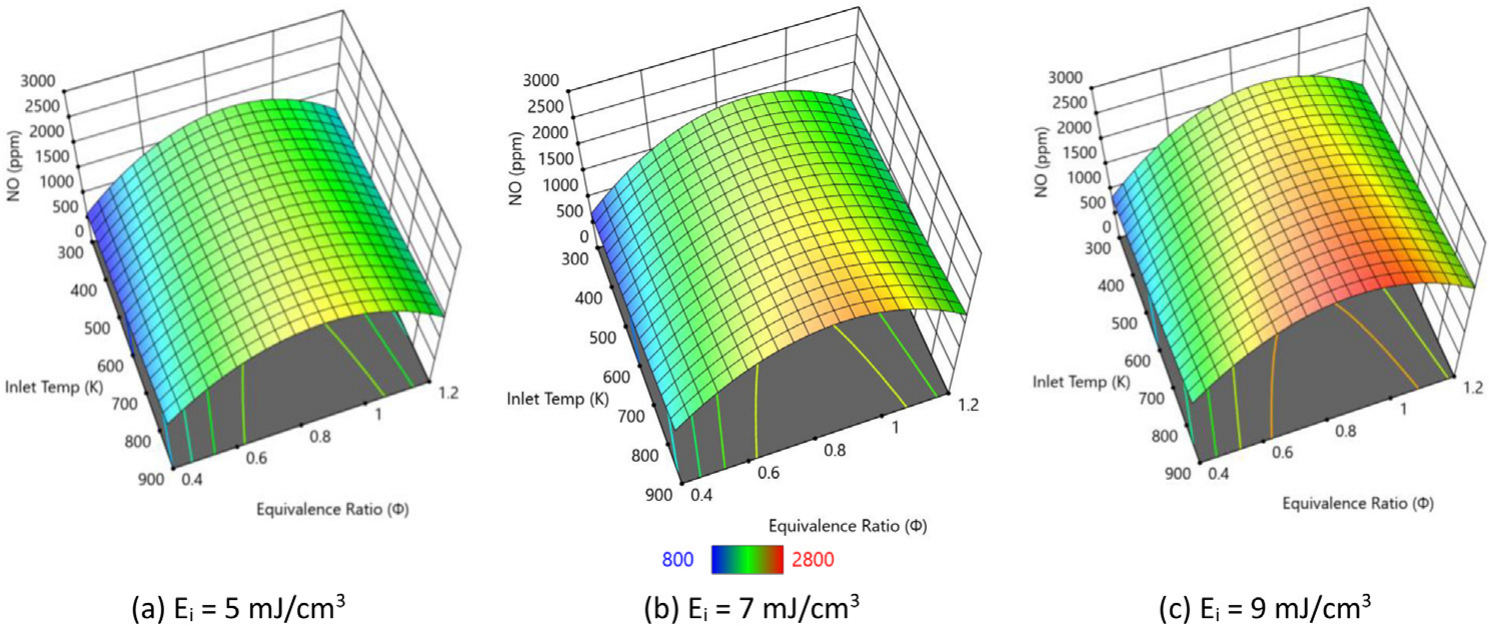
NO emissions at varying plasma energies for pure hydrogen at 1 atm.

Fig. 6 shows that increasing plasma pulse energy leads to a marked rise in ●NO emissions across the whole range of inlet temperatures and equivalence ratios. For all three energies, ●NO increases with inlet temperature and peaks near stoichiometric or slightly rich mixtures, consistent with stronger thermal and prompt NO formation at higher temperatures and higher radical concentrations. Comparing 5, 7, and 9 mJ/cm³, the ●NO surface shifts upward as plasma energy increases, indicating that the additional radicals and higher peak temperatures produced by stronger discharges intensify NO-forming pathways. The result highlights a trade-off: while higher plasma energies shorten ignition delay and enhance combustion, they also significantly promote ●NO formation in pure hydrogen at 1 atm, especially at high inlet temperatures and near-stoichiometric conditions.

### 3.3 Species profiles and production of key reaction rates

**Fig. 7:**
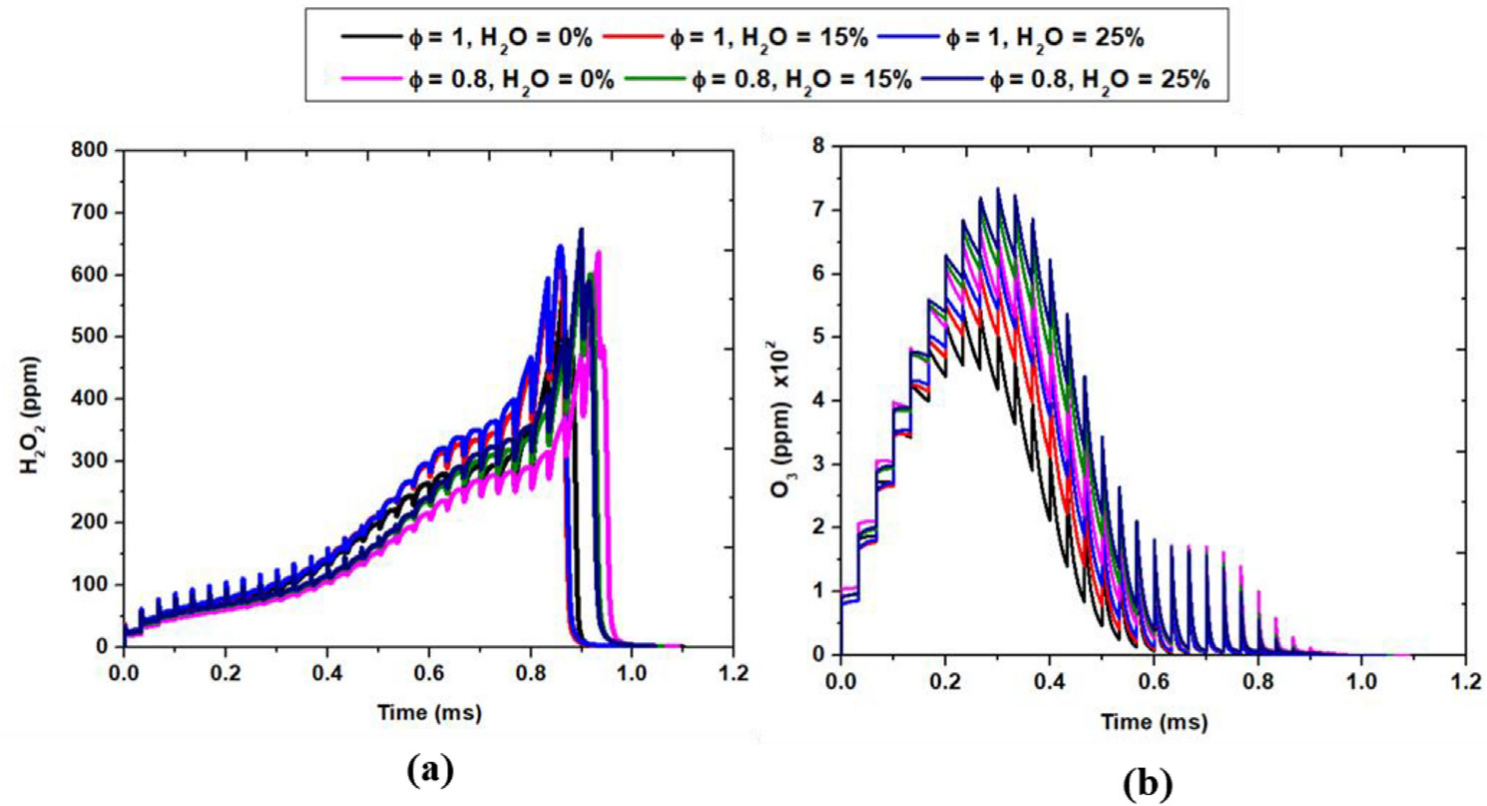
Temporal evolution of species profiles at two different equivalence ratios ϕ = 0.8 and ϕ = 1, with H₂O addition levels of 0 %, 15 %, and 25 %.

Fig. 7 provides a detailed temporal evolution of key species profiles (H_2_O_2_, and O_3_) under varying combustion conditions, specifically two equivalence ratios (ϕ = 0.8 and ϕ = 1) and three levels of H_2_O addition (0 %, 15 %, and 25 %). This allows for a comparative analysis of how these parameters influence radical formation, product generation, reactant consumption, and overall reaction progression over time.

H₂O₂ is an important intermediate species that directly influences ignition dynamics in H₂/H₂O mixtures under NRPD conditions. The temporal evolution of H₂O₂ reveals a distinct trend governed by both the equivalence ratio and the level of H₂O addition. As shown in Fig. 7a, H₂O₂ concentrations increase steadily during the early stages of plasma-assisted ignition, peaking just before the main heat release event. This behavior reflects the dominant role of H₂O₂ as a radical reservoir in low-temperature plasma-assisted combustion chemistry. The accumulation of H₂O₂ is primarily facilitated by the recombination of hydroperoxyl (HO₂) radicals through the reaction:

HO_2_+HO_2_→H_2_O_2_+O_2_

The presence of NRPD significantly enhances the production of HO₂ due to the rapid generation of ●O and ●OH radicals from electron impact dissociation of O₂ and H₂O. These radicals undergo three-body recombination, particularly in the presence of abundant third-body species such as H₂O, leading to the efficient formation of H₂O₂. As the level of H₂O dilution increases from 0 % to 25 %, the peak H₂O₂ concentrations are observed to rise. This can be attributed to two synergistic effects: first, water vapor acts as an effective third-body species that stabilizes H₂O₂ during its formation; and second, it suppresses the thermal decomposition of H₂O₂ by reducing the flame temperature and promoting chain termination reactions. At φ = 1.0, the effect is more pronounced due to a richer radical pool and higher local reactivity compared to the leaner φ = 0.8 case. These results highlight that water addition not only modulates the temperature and ignition delay but also actively influences the intermediate radical chemistry, particularly enhancing the accumulation of species like H₂O₂ that are critical to the propagation of plasma-initiated ignition fronts.

The temporal evolution of ozone (O₃), as illustrated in Fig. 7b, reflects the intricate coupling between plasma-induced radical production and post-discharge recombination chemistry. Ozone acts as a strong oxidizer and a radical initiator in nanopulsed plasma-assisted combustion. At early stages, O₂ molecules are excited and dissociate to form atomic oxygen (●O), which then reacts with O₂ to form O_3._

*O* + O_2_+M→O_3_+M

At lean condition ϕ = 0.8, this impact is clearer, because of higher O_2_ concentrations. O₃ is unstable and rapidly decomposes into highly reactive ●O atoms, which initiates chain-branching combustion reactions. With increasing levels of H₂O dilution (from 0 % to 25 %), the results indicate a substantial rise in peak O₃ concentrations. This enhancement is attributed to the dual role of H₂O: first, as an effective third-body collider that facilitates the stabilization of ozone molecules; and second, as a moderator of post-discharge temperatures, which reduces the rate of thermal decomposition of O₃ via:

O_3_+M→O_2_+*O* + M

Although O₃ itself is not a major heat-releasing species, its role as a long-lived oxidizing agent becomes critical in plasma-assisted combustion. It can act as a delayed radical source, thermally decomposing in downstream regions to reintroduce ●O and O₂, thus extending the radical pool and sustaining combustion in marginal conditions. These findings are consistent with the modeling and experimental work reported by Starik et al. [7], who highlighted the significant role of O₃ in enhancing ignition under nanosecond plasma discharge, particularly in the low-temperature regime. Moreover, the increased O₃ levels with water dilution suggest that controlled humidity or water injection strategies could be harnessed in future designs of plasma-assisted ignition systems to enhance radical generation efficiency while minimizing energy losses. This insight provides a pathway for optimizing plasma-flame coupling in low-temperature and lean combustion systems.

**Fig. 8:**
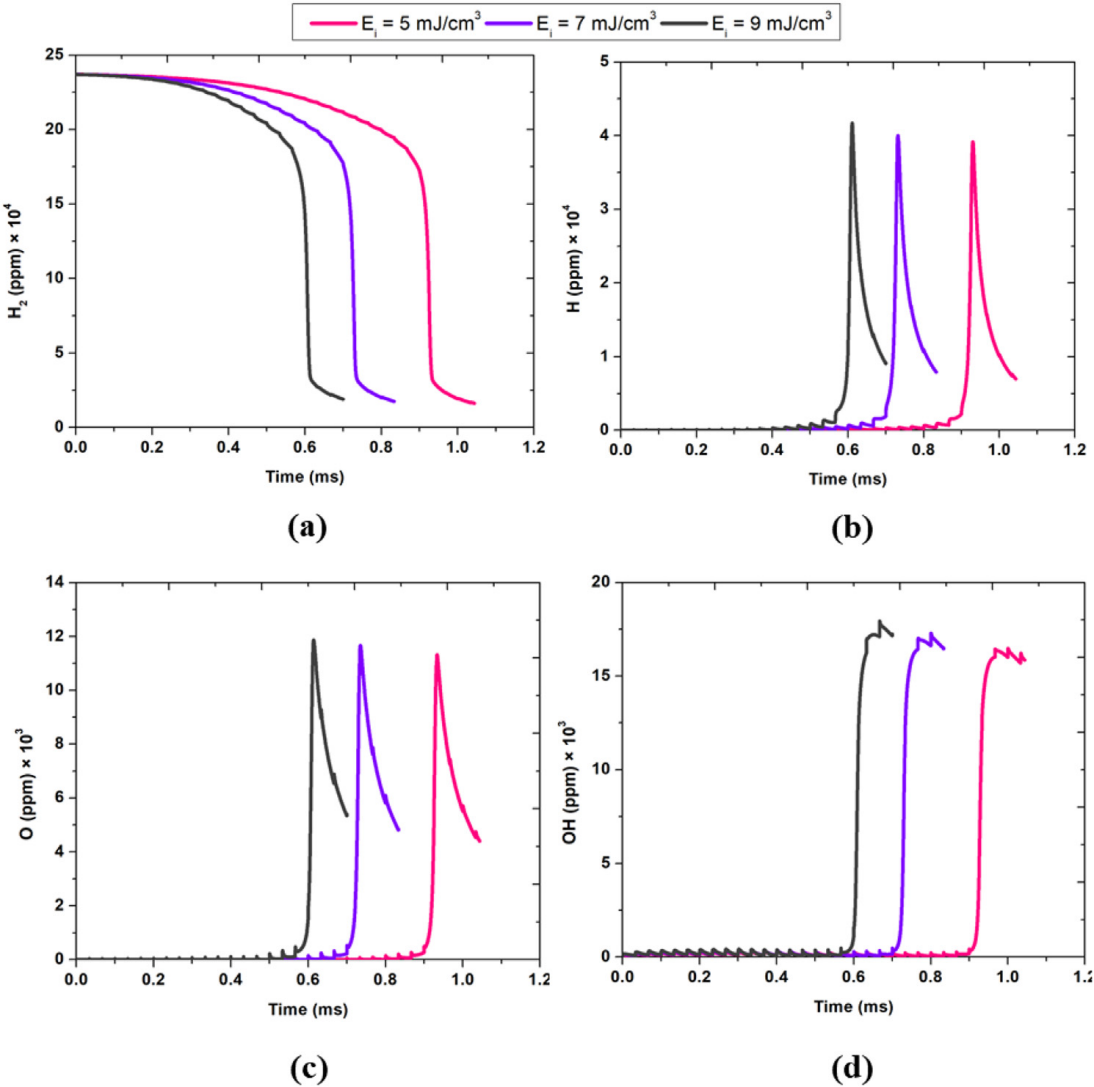
Temporal evolution of (a) H₂, (b) ●H, (c) ●O, and (d) ●OH species at a fixed H₂O concentration of 25 %, under ambient temperature and pressure conditions. Results are shown for three different plasma energy densities: 5, 7, and 9 mJ/cm³.

Fig. 8 illustrates the profound influence of plasma energy density on the temporal evolution of key species H₂, ●H, ●O, and ●OH—at a fixed water concentration of 25 % under ambient conditions. As plasma energy density increases from 5 to 9 mJ/cm³, the ignition process is markedly accelerated, as evidenced by the earlier and more rapid decline in H₂ concentration (Fig. 8a) and the corresponding earlier and higher peaks in ●H, ●O, and ●OH radical concentrations (Figs. 8b–d). This trend is attributed to the greater abundance of energetic electrons at higher energy densities, which enhances the rates of electron impact dissociation of H₂O and H₂, thereby promoting the swift formation of reactive radicals. The radicals ●H, ●O, and ●OH all exhibit sharp, temporally shifted peaks, with both their appearance and disappearance occurring sooner as energy density increases. Notably, the magnitude of these radical peaks also rises with increasing energy input, indicating more vigorous plasma-driven chemistry. These results collectively demonstrate that higher plasma energy densities not only expedite the onset of ignition but also intensify radical production, fundamentally altering the reaction environment and potentially improving the efficiency of plasma-assisted combustion processes. The observed behaviours underscore the critical role of precise energy control in optimizing radical generation and ignition timing in plasma-activated systems.

**Fig. 9:**
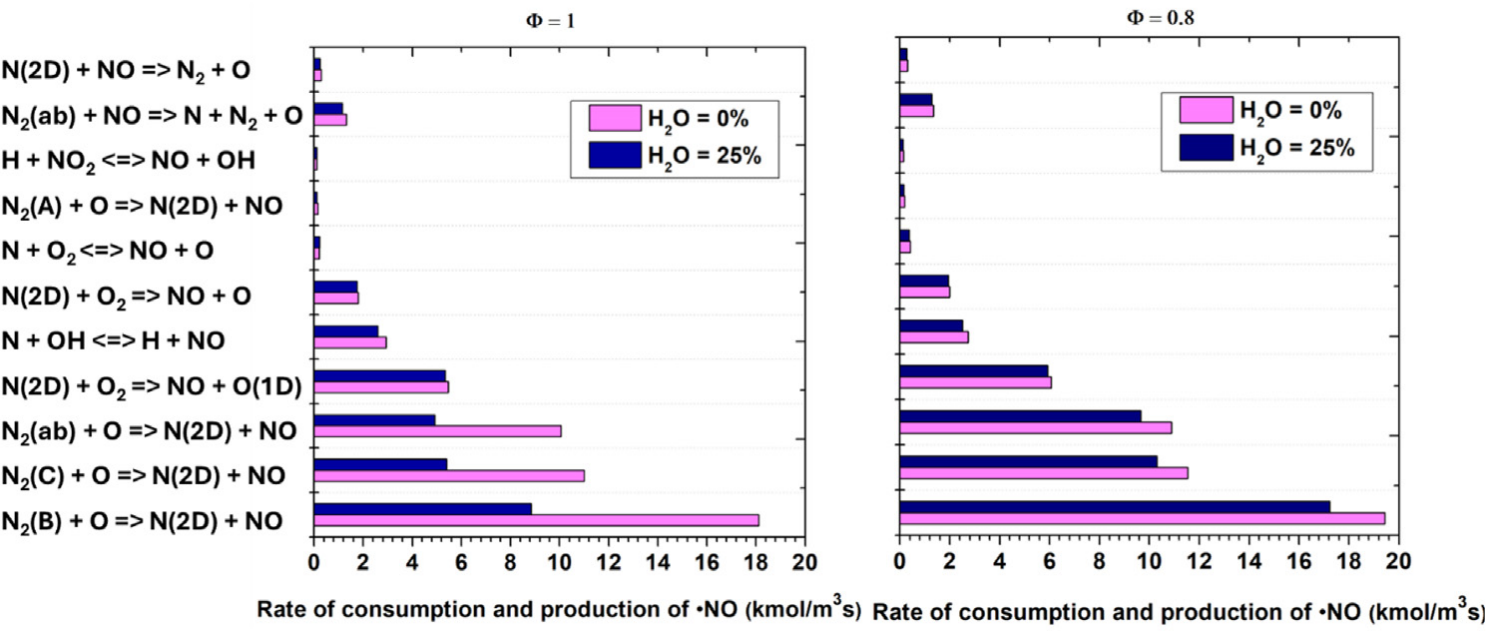
Rate of ●NO production and consumption for equivalence ratios φ = 1.0 and φ = 0.8, with and without water addition.

Fig. 9 presents a comparative analysis of the rates of ●NO production and consumption via key elementary reactions under stoichiometric (φ = 1.0) and lean (φ = 0.8) conditions, with and without water addition. The results reveal that water vapor (25 % H₂O) substantially suppresses both ●NO formation and destruction pathways compared to the dry case (0 % H₂O) for both equivalence ratios. This suppression is particularly pronounced under lean conditions, where the overall rates are lowest. The dominant ●NO production channels are reactions involving electronically excited nitrogen species—specifically, N₂(B), N₂(C), and N₂(ab)—reacting with atomic oxygen to form ●NO and higher excited N₂ states. Water addition reduces the rates of these plasma-specific pathways, primarily by quenching excited nitrogen states and decreasing the availability of atomic oxygen through enhanced radical recombination and energy partitioning into non-reactive channels. The primary ●NO consumption pathway, N₂(D) + NO → N₂ + *O*, is similarly diminished in the presence of water. The overall higher rates observed at φ = 1.0 reflect the greater abundance of reactive intermediates and higher flame temperatures under stoichiometric conditions. These findings highlight the dual impact of water vapor: it not only moderates the formation of ●NO by limiting the pool of excited and atomic species but also dampens ●NO removal processes, with the net effect being a substantial reduction in NO turnover, especially in lean, water-rich environments. This underscores the critical role of water in controlling plasma-assisted NOx chemistry and provides valuable insight for optimizing emissions in plasma-activated combustion systems.

**Fig. 10:**
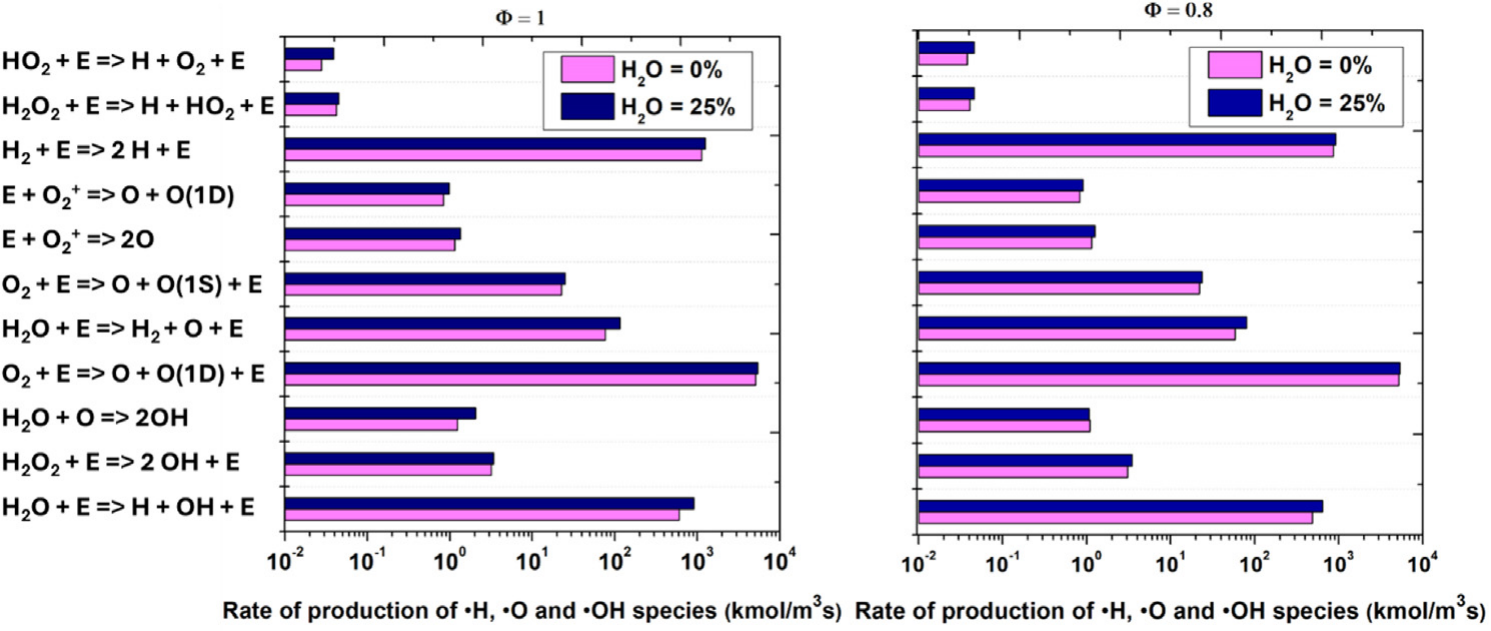
Rate of production of active particles ●H, ●O, and ●OH for equivalence ratios φ = 1.0 and φ = 0.8, with and without water addition.

Fig. 10 provides a comprehensive comparison of the production rates of key reactive species ●H, ●O, and ●OH across major plasma-induced reactions for both stoichiometric (φ = 1.0) and lean (φ = 0.8) conditions, with and without water addition. The data reveal that introducing 25 % H₂O enhances the rates of reactions involving direct electron impact dissociation of water, such as H₂*O* + *e*⁻ → *H* + OH + *e*⁻ and H₂*O* + *e*⁻ → OH + *H* + *e*⁻, for both equivalence ratios. This effect is most pronounced for the generation of ●H and ●OH radicals, where the presence of water provides abundant dissociation targets, resulting in blue bars that significantly exceed their dry-case (pink) counterparts. In contrast, the rates of reactions not directly involving water, such as O₂ + *e*⁻ → *O* + *O* + *e*⁻ and *E* + *O*₂(A*) → *O* + *O*(¹D), remain largely unchanged or are slightly reduced with water addition, indicating that water’s primary impact is through its own dissociation rather than by enhancing the dissociation of other species. Notably, the overall radical production rates are higher at φ = 1.0 than at φ = 0.8, reflecting the greater availability of reactants and a more robust radical pool in stoichiometric mixtures. However, in lean conditions, the relative contribution of water-driven pathways becomes even more significant, as baseline radical generation is otherwise limited. These findings underscore that water vapor in plasma environments is not merely a diluent but an active participant that fundamentally shifts the chemistry toward H₂O-driven radical generation, especially for H and OH. The results highlight the importance of both mixture stoichiometry and water content in improving plasma-assisted ignition and combustion, as they govern the efficiency and dominant mechanisms of active species production.

**Fig. 11:**
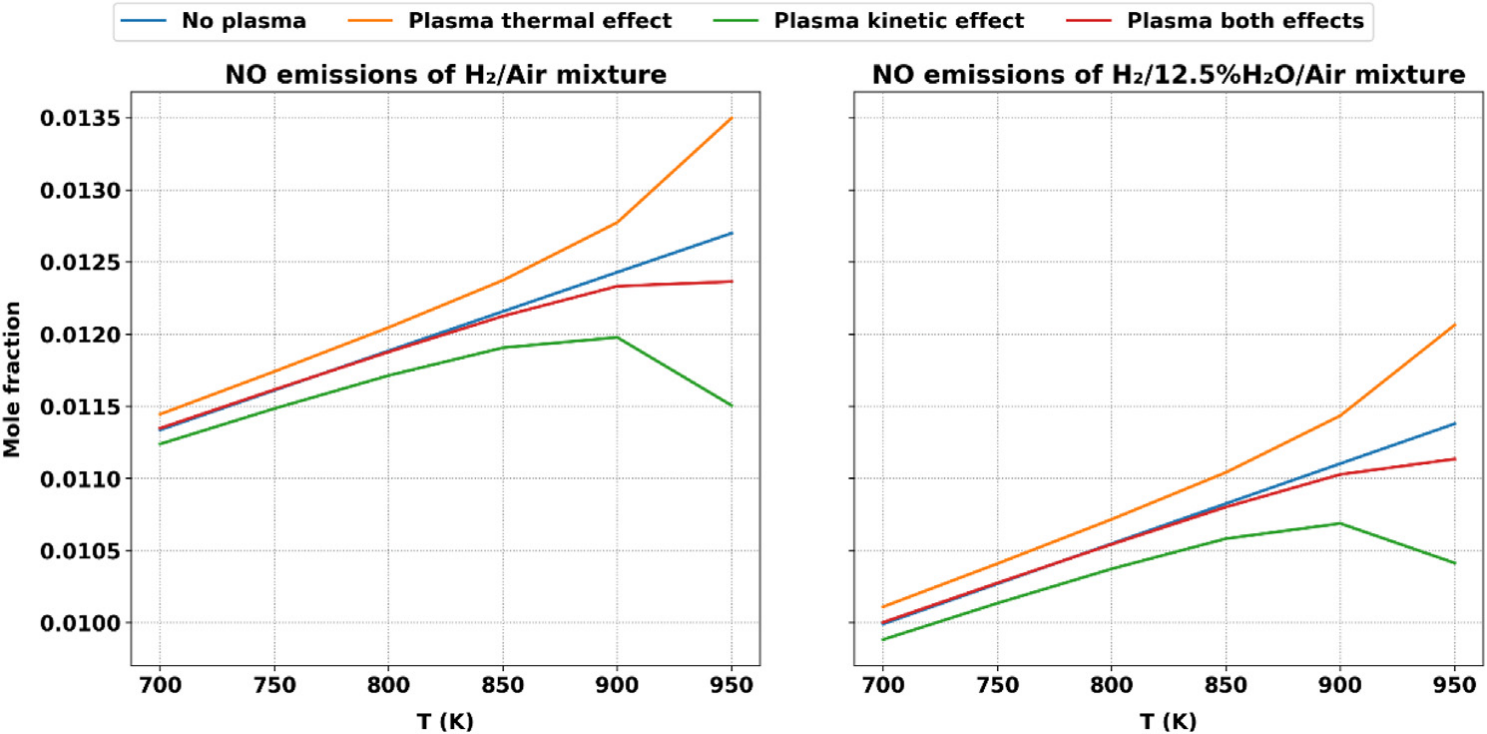
Comparative Analysis of ●NO Emission Values at Different Inlet Temperatures for H₂/Air and H₂/12.5 % H₂O/Air Mixtures, Highlighting Plasma Thermal and Kinetic Effects.

Fig. 11 presents a comparative analysis of nitric oxide (●NO) emissions at different inlet temperatures for two fuel mixtures: H₂/Air and H₂/12.5 % H₂O/Air, examining the effects of plasma applications across thermal and kinetic domains.

In the baseline scenario without plasma (blue line), ●NO emissions rise with increasing temperature due to the inherent thermal nature of ●NO formation. Plasma thermal effects, (orange line), further enhance ●NO emissions as elevated temperatures drive the formation of ●NO through conventional thermal pathways.

Conversely, the plasma kinetic effects, (green line), play a transformative role in reducing ●NO emissions across both fuel mixtures. Plasma-induced kinetic enhancements involve the generation of active radicals and excited species that alter chemical kinetics, significantly weakening or rerouting the pathways responsible for ●NO formation.

The inclusion of water vapor in the H₂/12.5 % H₂O/Air mixture amplifies these kinetic effects, as seen in the right plot. Water molecules contribute to the generation of additional radical species, fostering reaction pathways that further curtail ●NO production. The synergy between water vapor and plasma kinetic effects highlights a powerful interaction that promotes cleaner combustion processes by enhancing radical-driven reactions and reducing pollutant formation. Overall, the comparative analysis underscores the dominant role of plasma kinetic effects and their potential to counteract the NO-promoting tendencies of thermal effects, particularly when augmented by water vapor.

## 4 Numerical Methods

This study employs ChemPlasKin, a zero-dimensional solver optimized for simulating chemical kinetics in non-equilibrium plasma environments, effectively integrating plasma and combustion kinetics in a unified framework. ChemPlasKin uses external libraries like Cantera for thermodynamics, CppBOLOS for electron Boltzmann equation solutions, muParser for complex rate expressions, and CVODES for solving stiff ODEs, enabling detailed time-resolved predictions of species concentrations and gas temperature. A novel heat loss model for nanosecond repetitively pulsed discharges is incorporated. The kinetic mechanism integrates reactions from literature with electron-impact cross sections from LXCat, encompassing plasma, neutral, ionization, quenching, and recombination processes, with a detailed focus on water vapor plasma reactions. Design of Experiments (DOE) was employed using Stat-Ease Design-Expert software to systematically vary key input parameters temperature, pressure, equivalence ratio, plasma energy, and water fraction generating a comprehensive matrix for robust modelling of plasma-water vapor synergistic effects on ignition delay, flame temperature, and NO_x_ emissions. Statistical validations ensured model accuracy and significance of interaction effects, supporting a thorough exploration of parameter space.

## Limitations

The model applied in this study is primarily a zero-dimensional chemical kinetics solver designed for detailed plasma-chemical interactions and combustion kinetics coupling. While it captures the intricate chemical pathways and plasma effects on ignition and reaction rates accurately, it inherently lacks spatial resolution and fluid dynamic coupling. Consequently, phenomena like flame flashbacks and local radical concentration gradients, which are governed by spatial transport, diffusion, and complex flow interactions, are not directly simulated in the current work.

## Ethics Statement

The authors followed generally expected standards of ethical behavior in scientific publishing throughout article construction*.*

## CRediT Author Statement

**Ghazanfar Mehdi:** Writing – review & editing, Writing – original draft, Validation, Software, Methodology, Investigation, Formal analysis, Conceptualization. **Eljas Almusa**: Writing – review & editing, Data curation. **Mihiran Pathmika Galagedarage Don**: Writing – review & editing, Software, Data curation. **Ossi Kaario**: Writing – review & editing, Supervision, Funding acquisition, Conceptualization. **Zubair Ali shah**: Writing – review & editing, Data curation. **Muhammad Basit Chandio:** Writing – review & editing, Data curation. **Maria Grazia De Giorgi**: Writing – review & editing, Supervision, Conceptualization.

## Data Availability

Mendeley DataDataset of Numerical assessment on the combined effects of non-thermal plasma and water addition in hydrogen combustion (Original data)

## Appendix Supplementary materials

Image, application 1
