# Supplementary material for: Dataset of numerical assessment on the combined effects of non-thermal plasma and water addition in hydrogen combustion
Source: Data Brief. 2025 Dec 19;64:112405. doi: 10.1016/j.dib.2025.112405 (PMC12813465; doi:10.1016/j.dib.2025.112405)

1. Effects of different inlet pressures using fixed plasma energy for pure hydrogen.

| 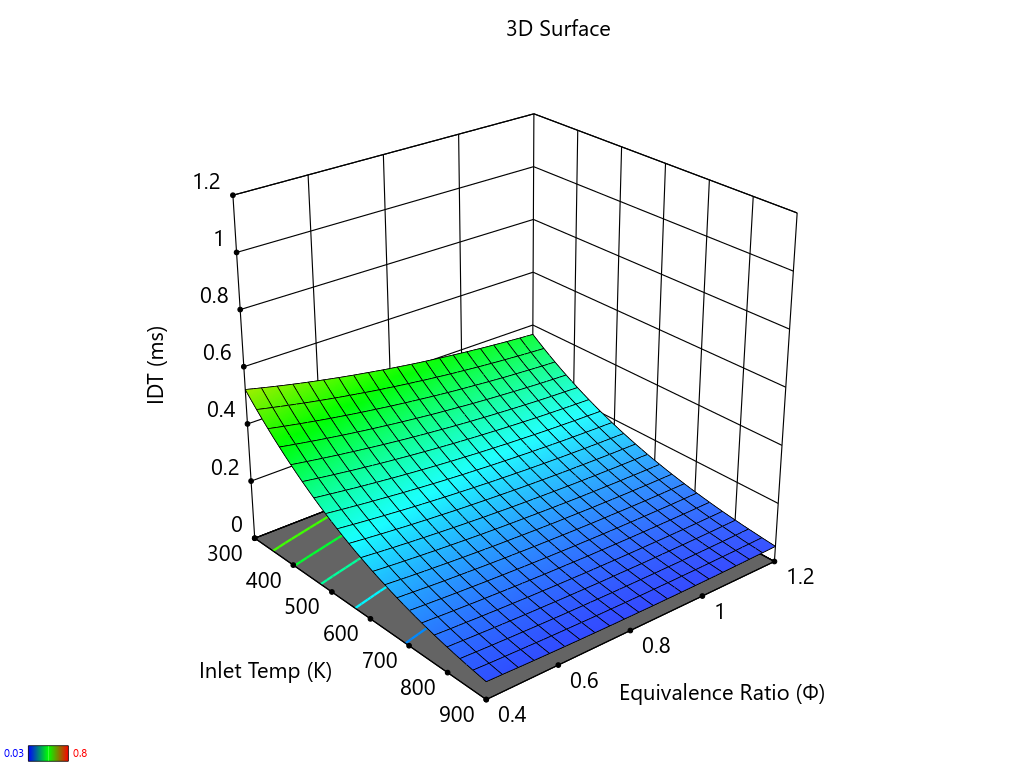 | 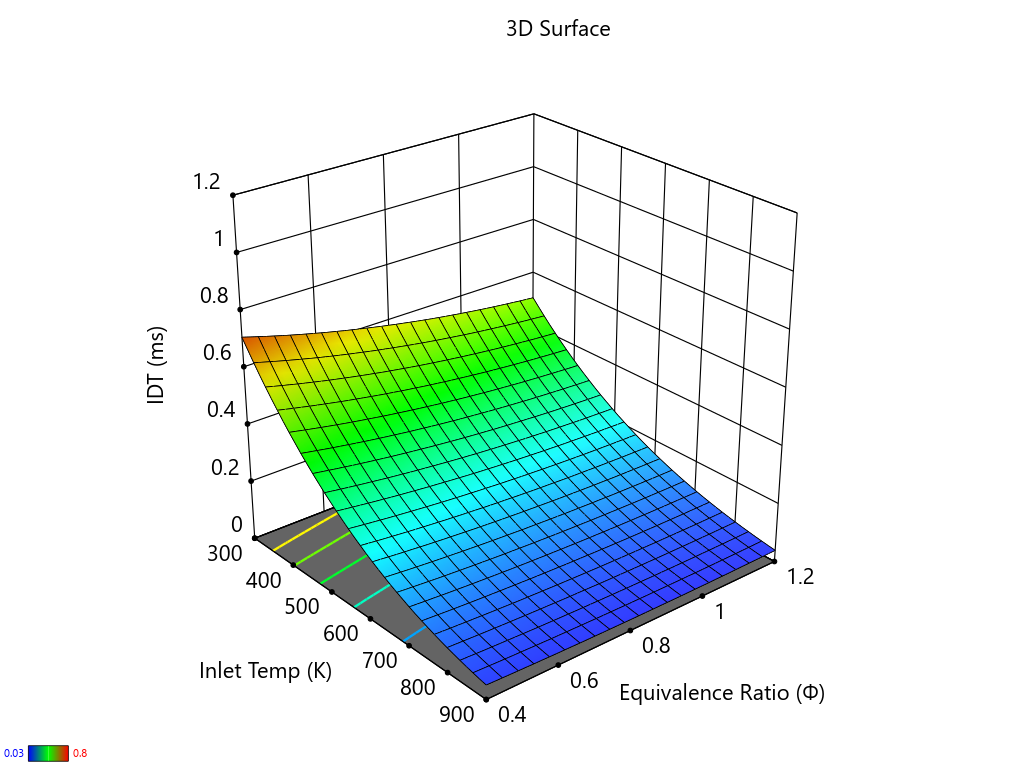 | 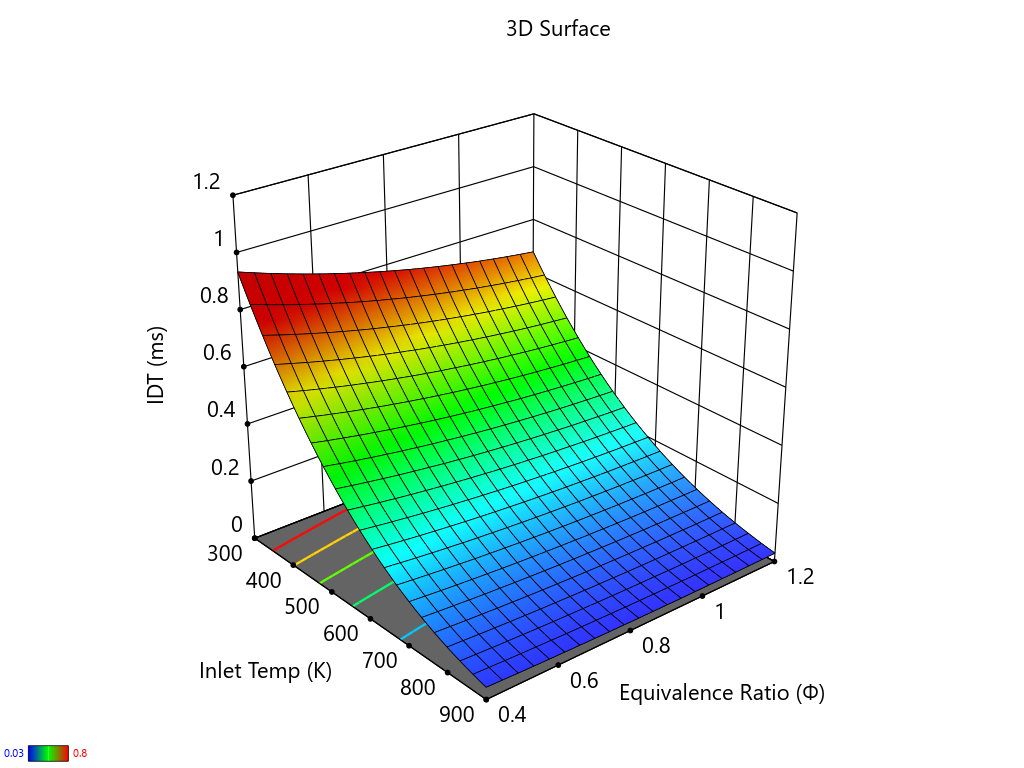 |
| --- | --- | --- |
|  | 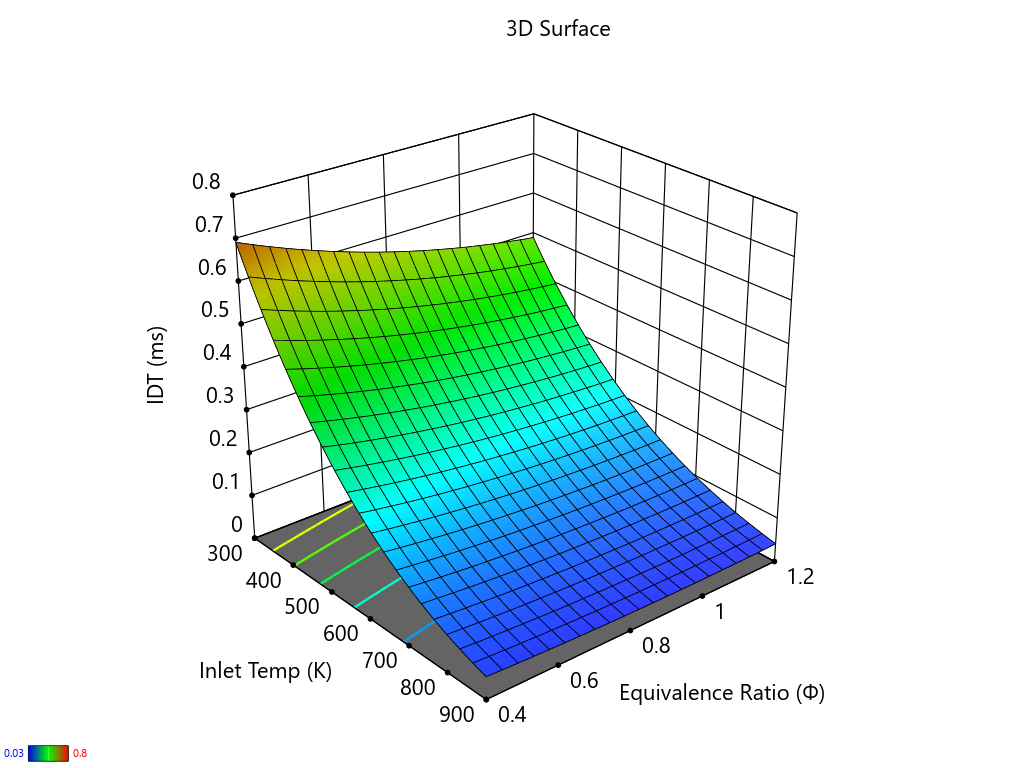 |  |
| (a) P= 0.5 atm | (b) P= 1 atm | (c) P= 1.5 atm |

Figure: IDT variation with different pressure inlets for pure hydrogen at E_i_ = 9 mJ/cm^3^.

| 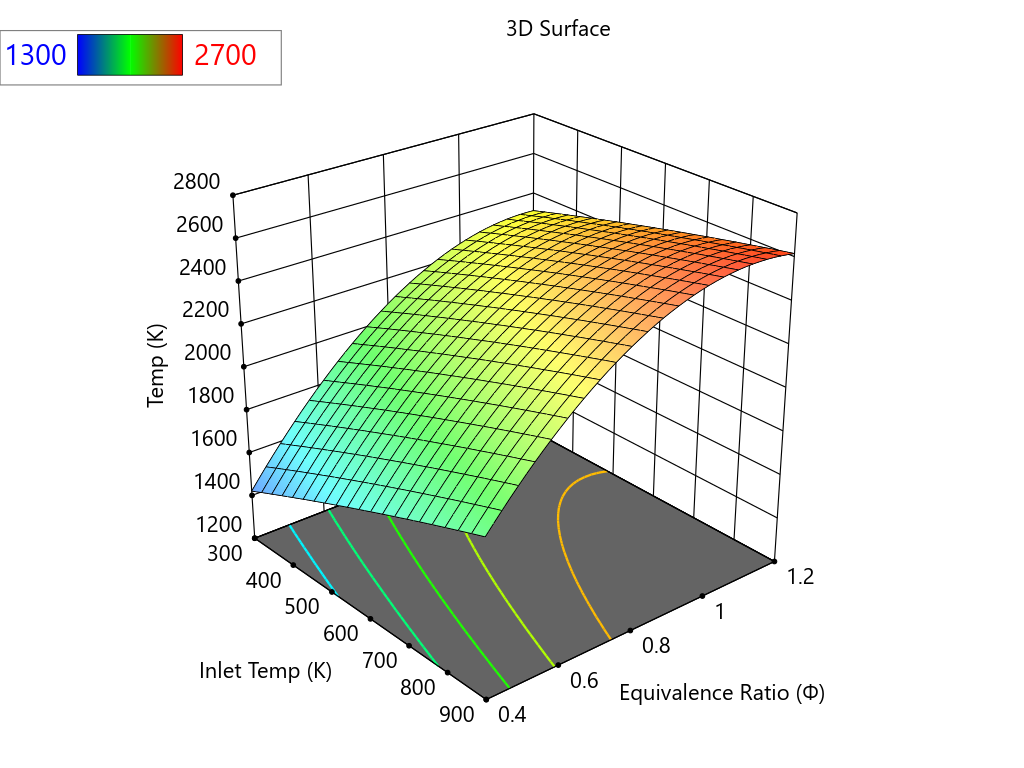 | 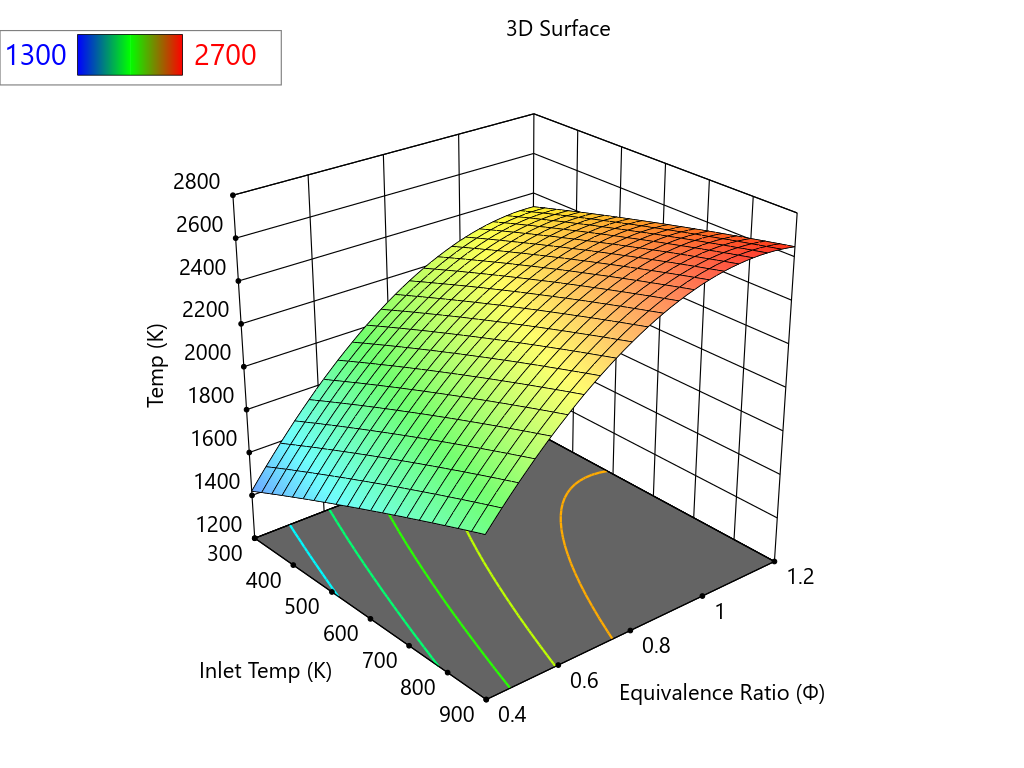 | 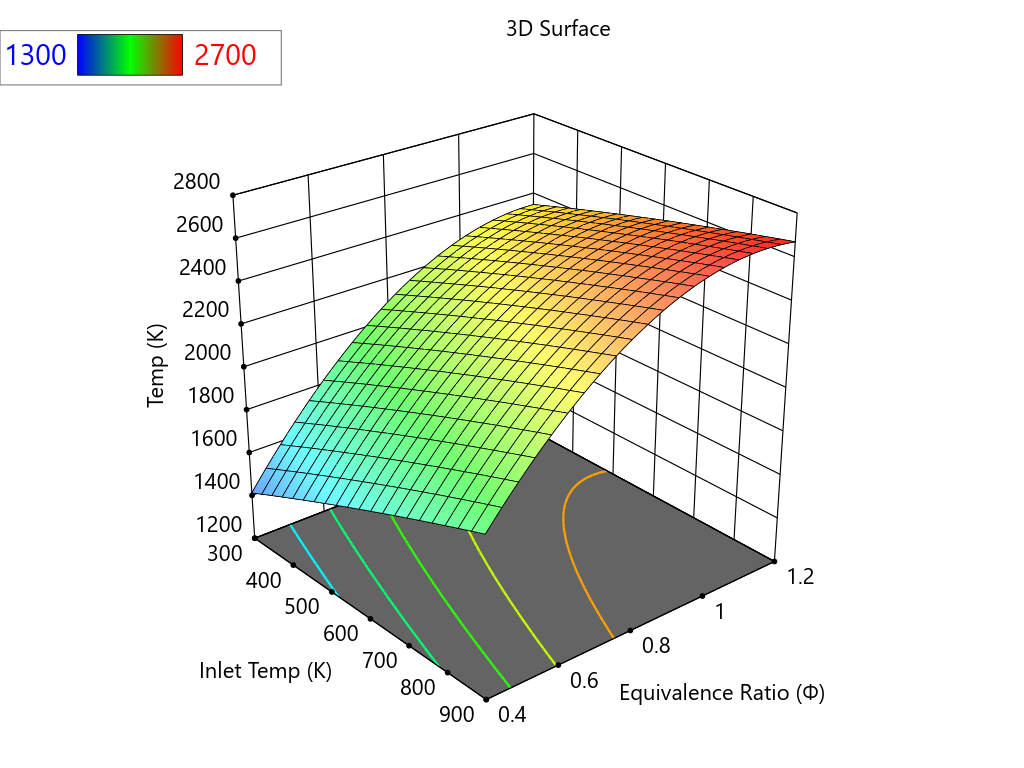 |
| --- | --- | --- |
|  | 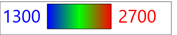 |  |
| (a) P= 0.5 atm | (b) P= 1 atm | (c) P= 1.5 atm |

Figure. Temperature changes with different pressure inlets for pure hydrogen at E_i_ = 9 mJ/cm^3^.

| 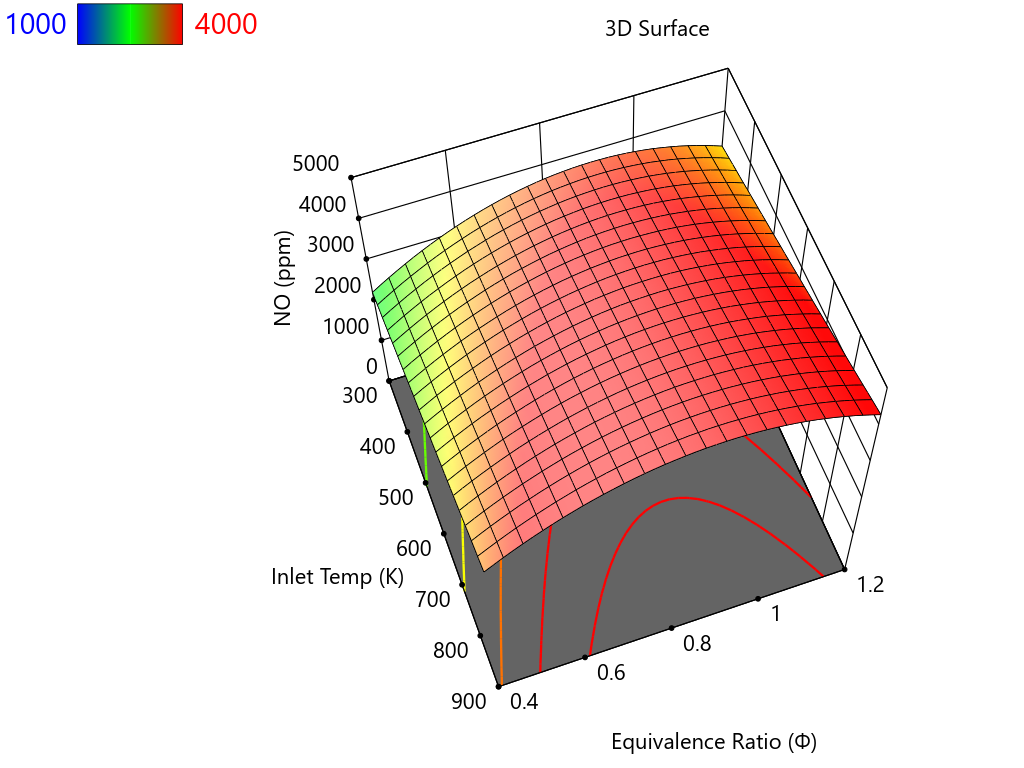 | 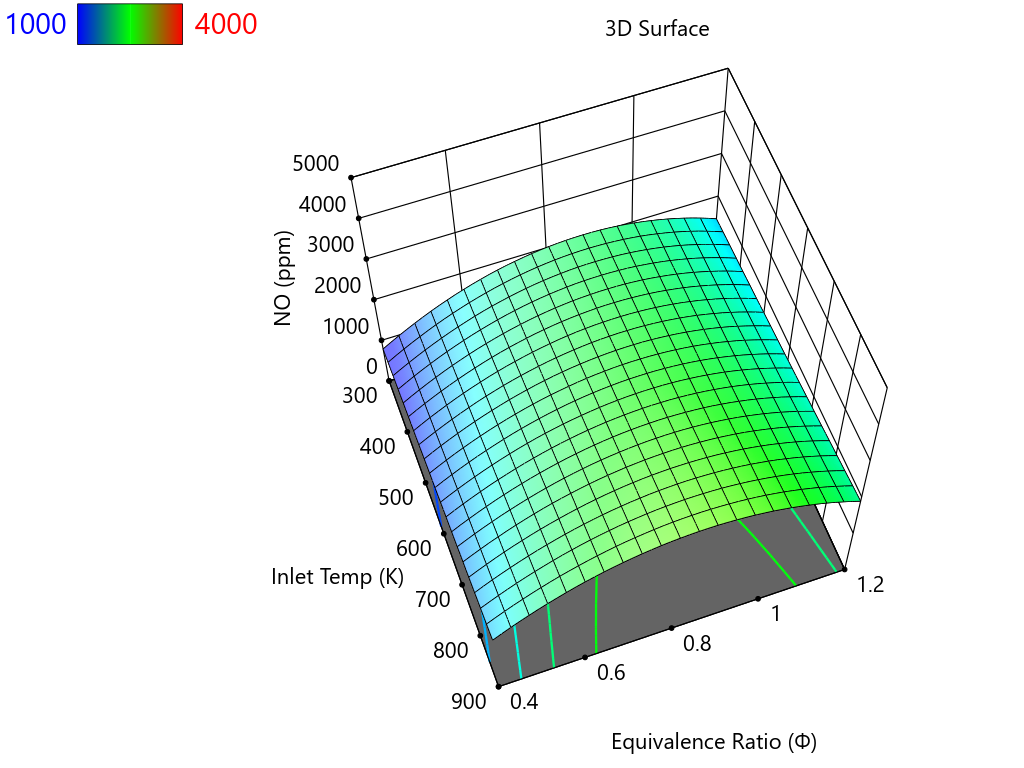 | 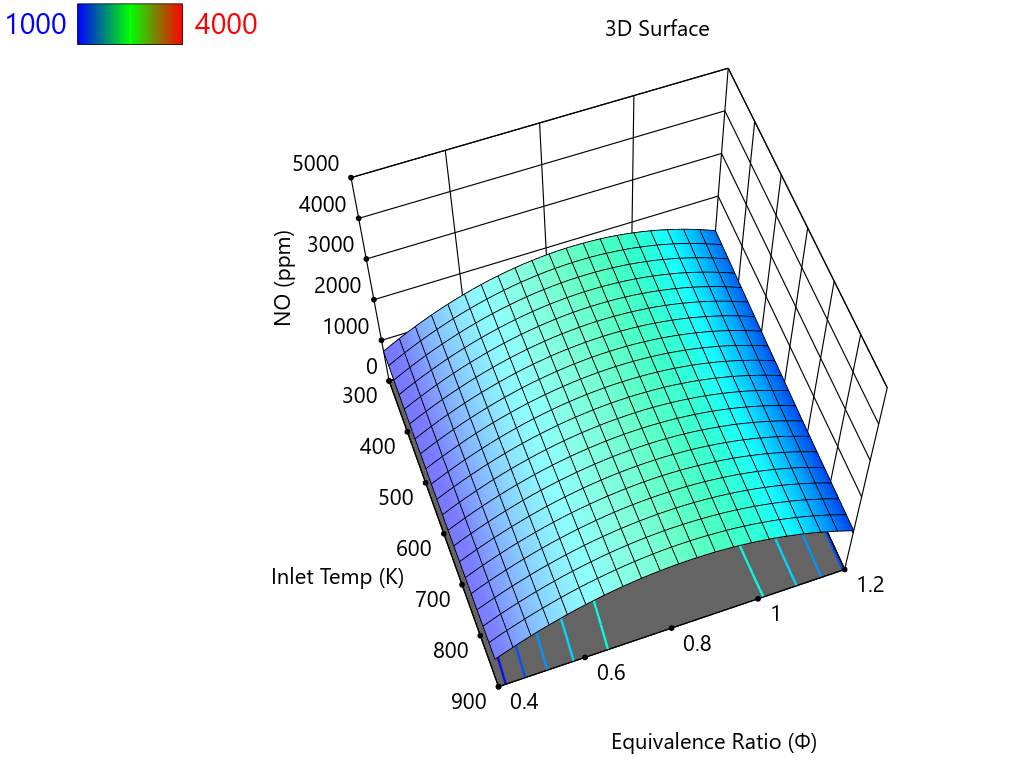 |
| --- | --- | --- |
|  | 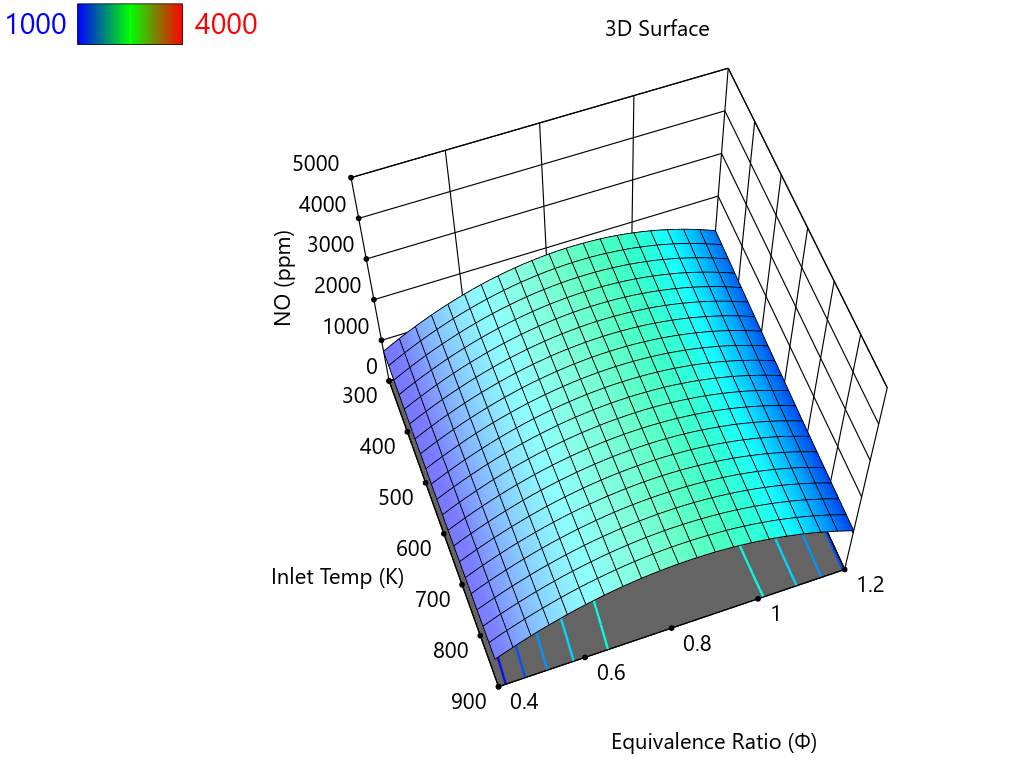 |  |
| (a) P= 0.5 atm | (b) P= 1 atm | (c) P= 1.5 atm |

Figure. NO emissions at varying pressure inlets for pure hydrogen at E_i_ = 9 mJ/cm^3^.

| 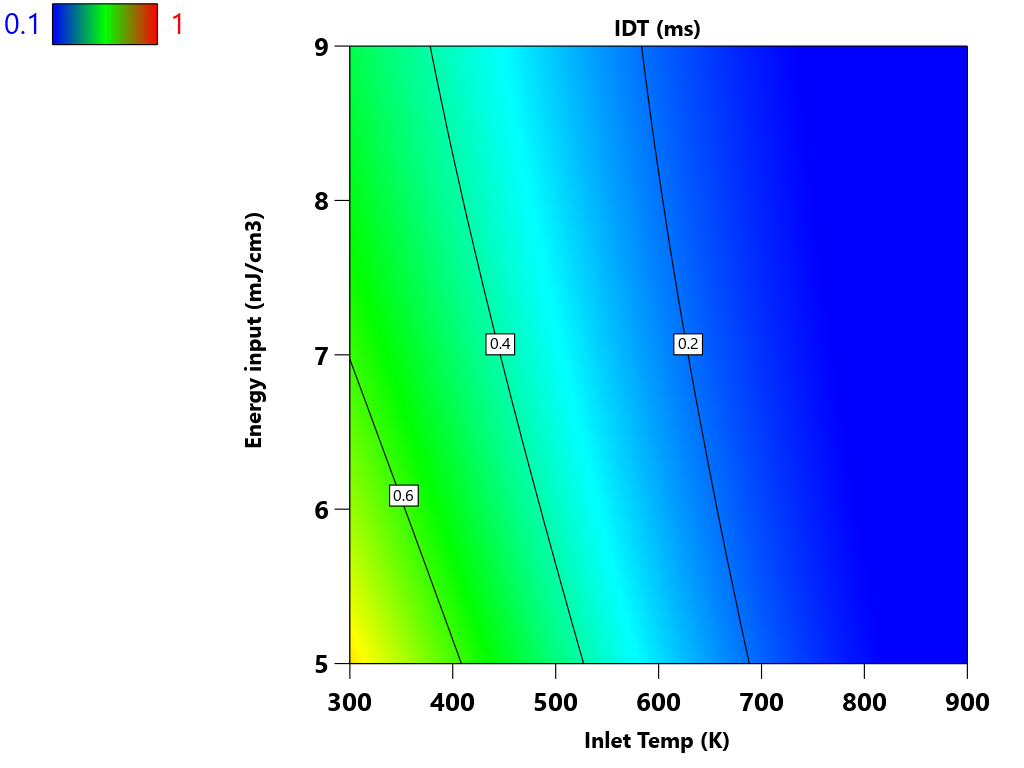 | | |
| --- | --- | --- |
| 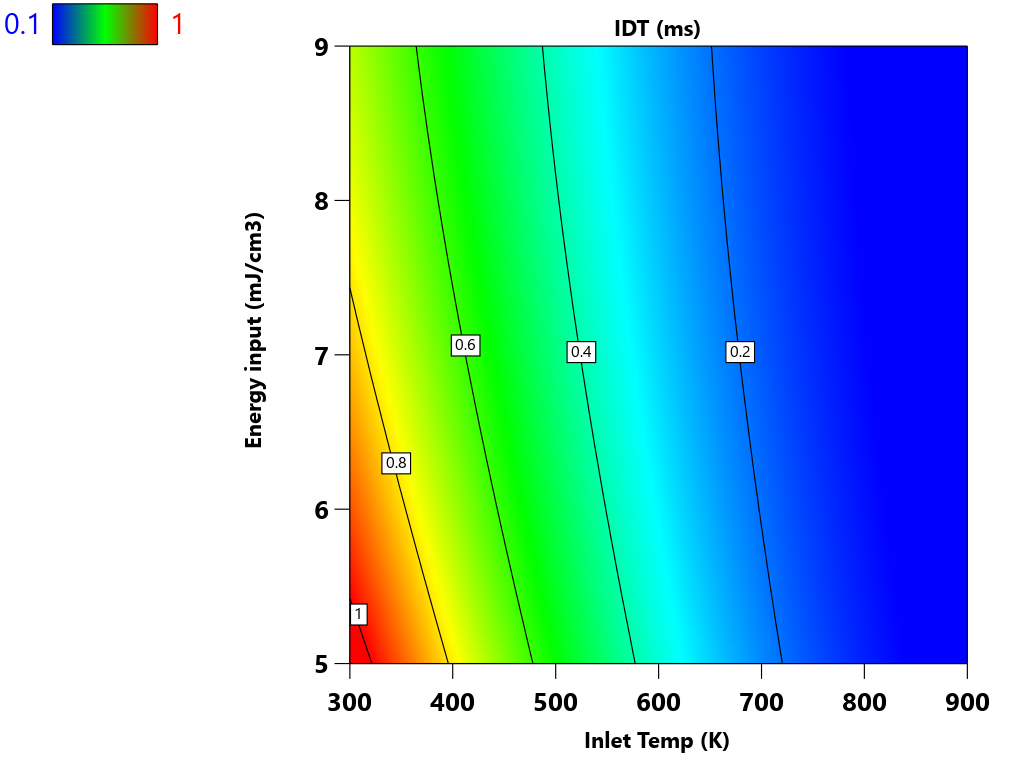 | 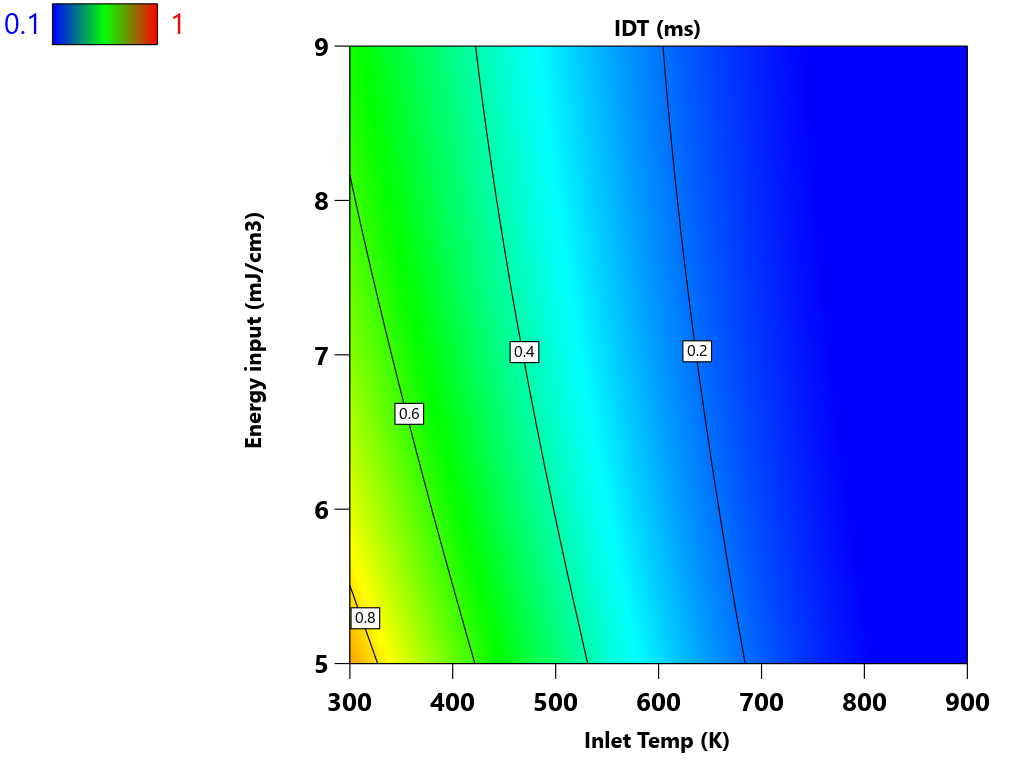 | 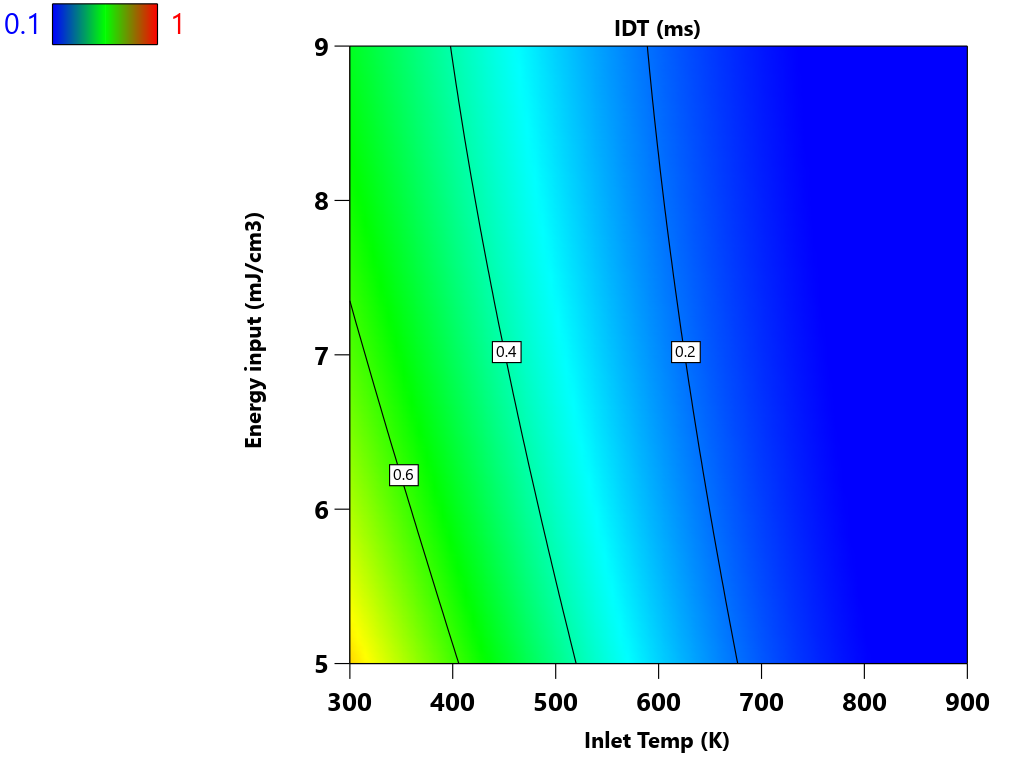 |
| (a) Φ= 0.4, H20 = 0% | (b) Φ= 0.8, H20 = 0% | (c) Φ= 1.2, H20 = 0% |
| 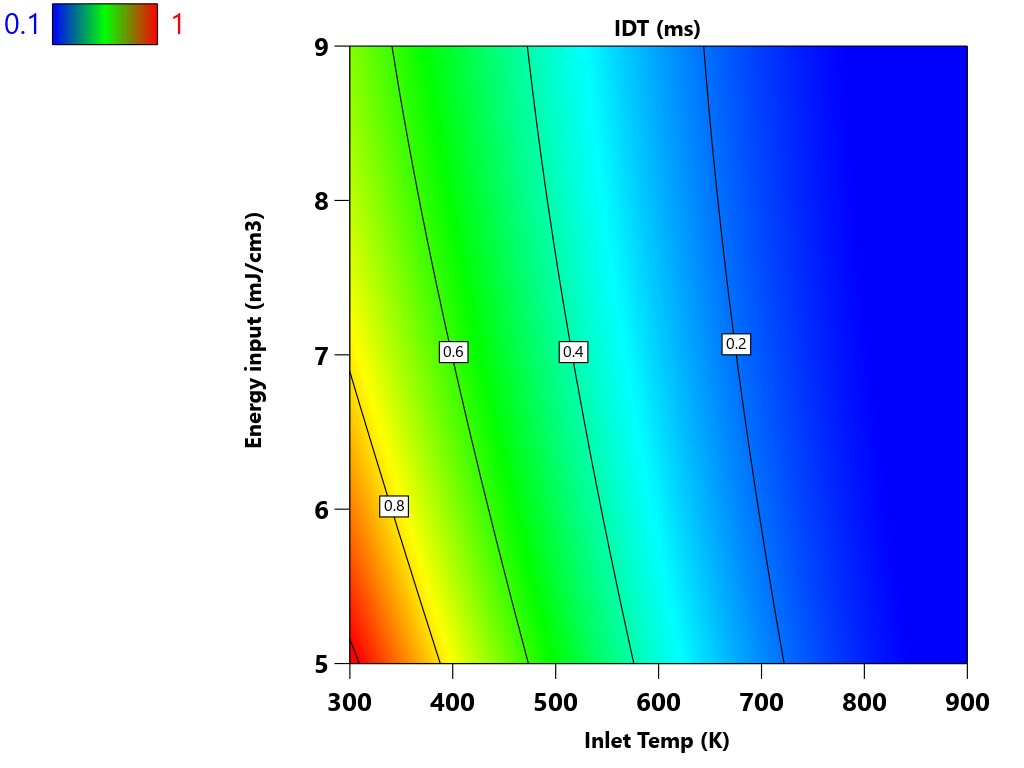 | 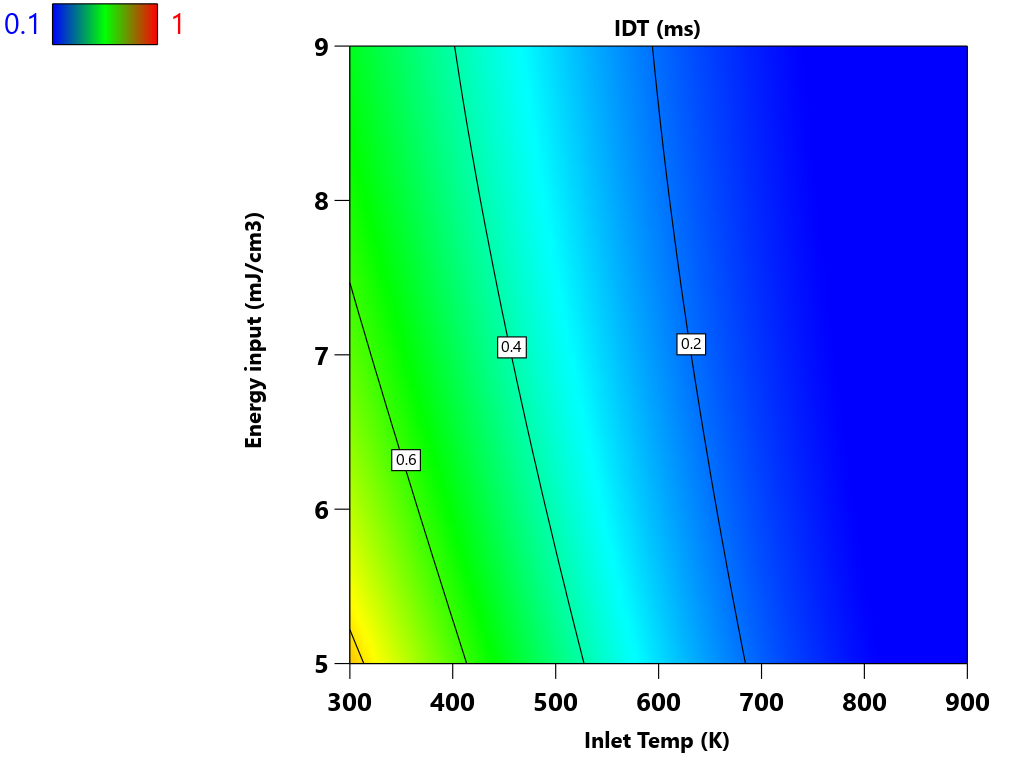 | 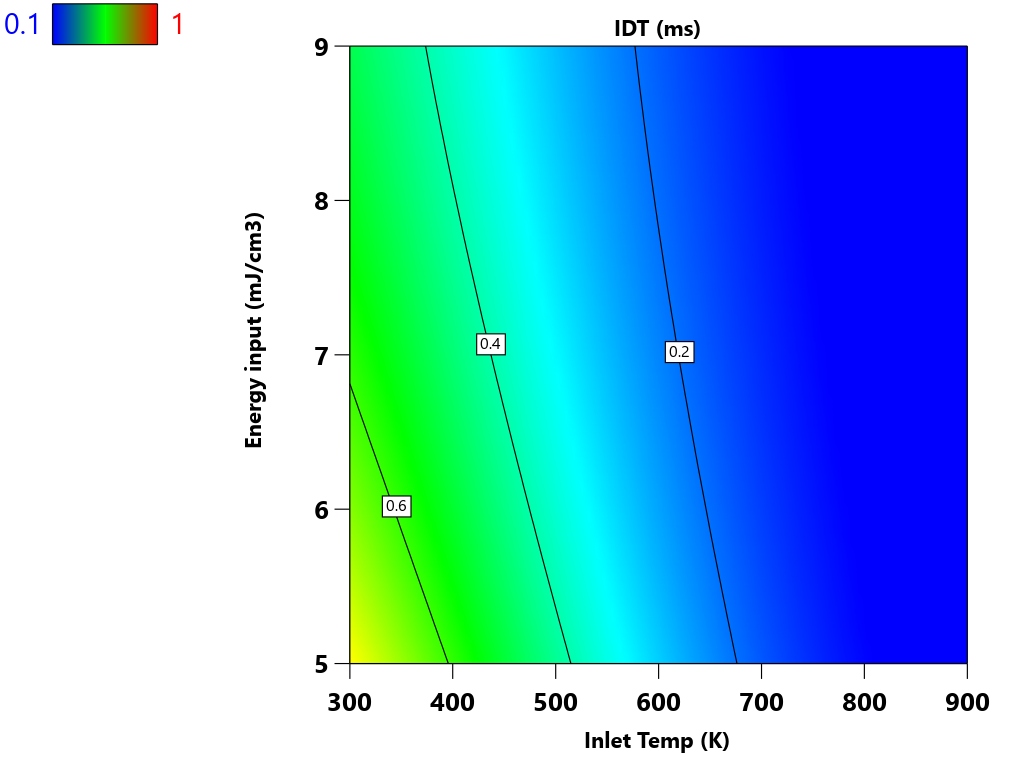 |
| (d) Φ= 0.4, H20 = 15% | (e) Φ= 0.8, H20 = 15% | (f) Φ= 1.2, H20 = 15% |
| 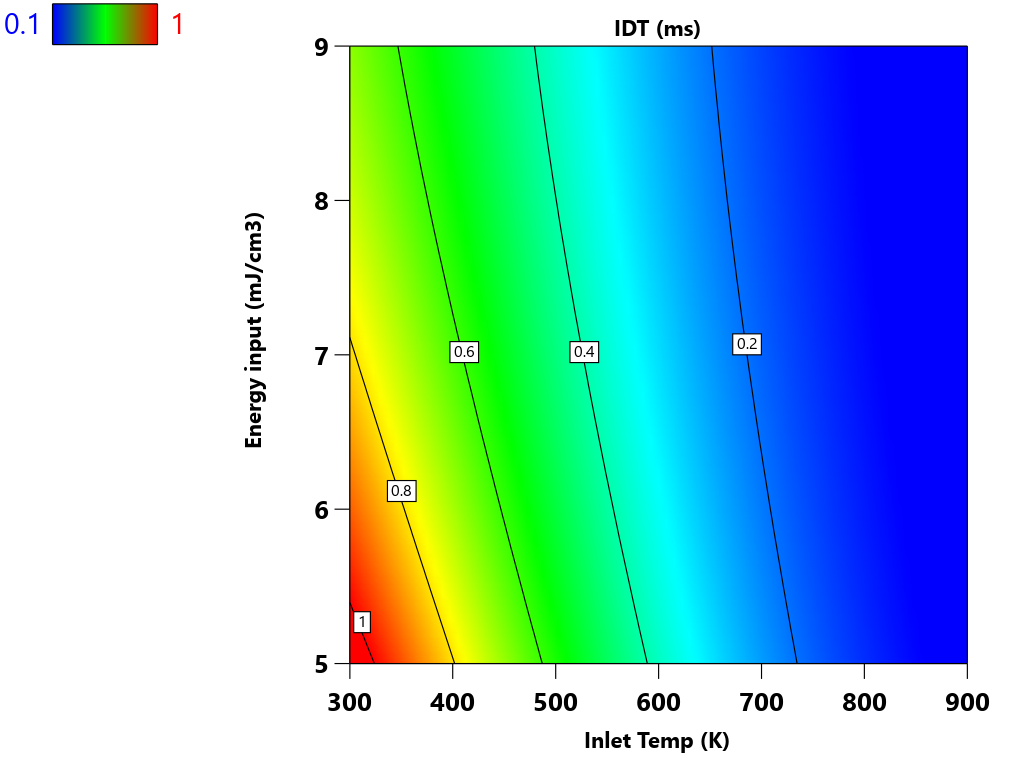 | 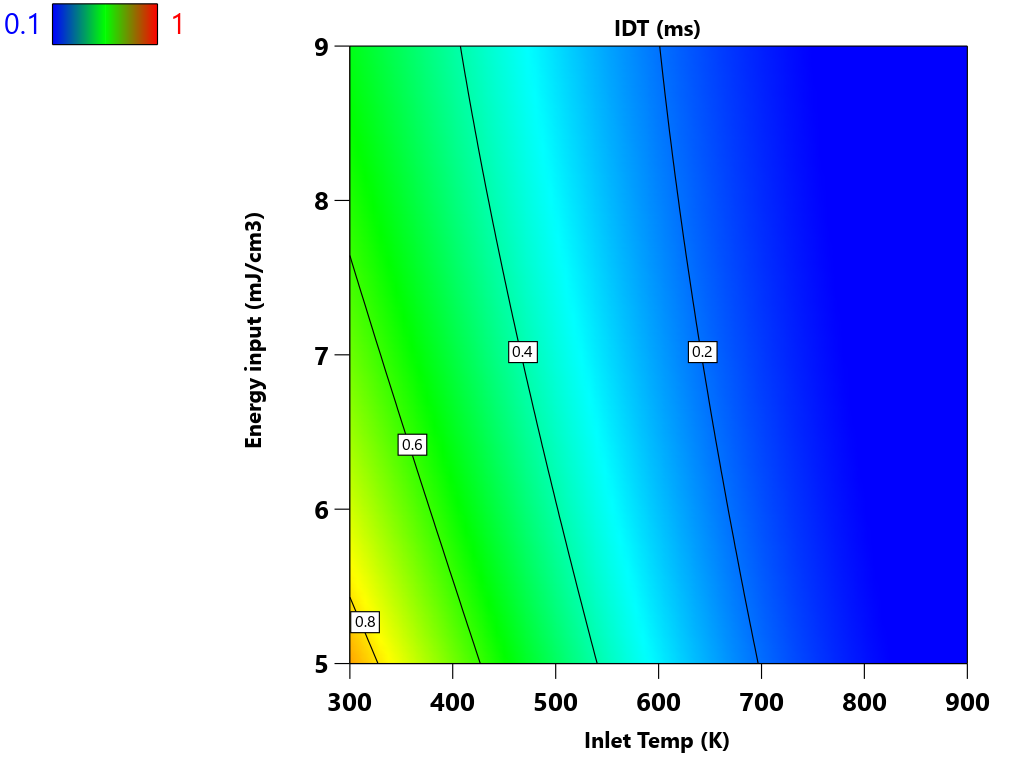 | 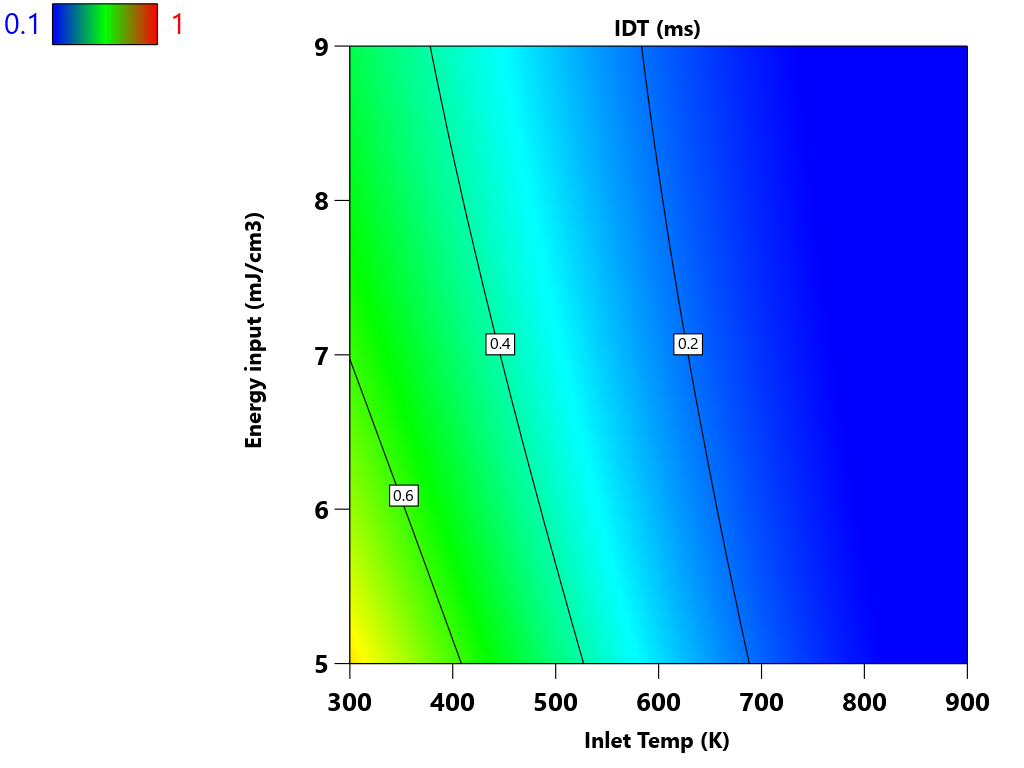 |
| (g) Φ= 0.4, H20 = 25% | (h) Φ= 0.8, H20 = 25% | (i) Φ= 1.2, H20 = 25% |


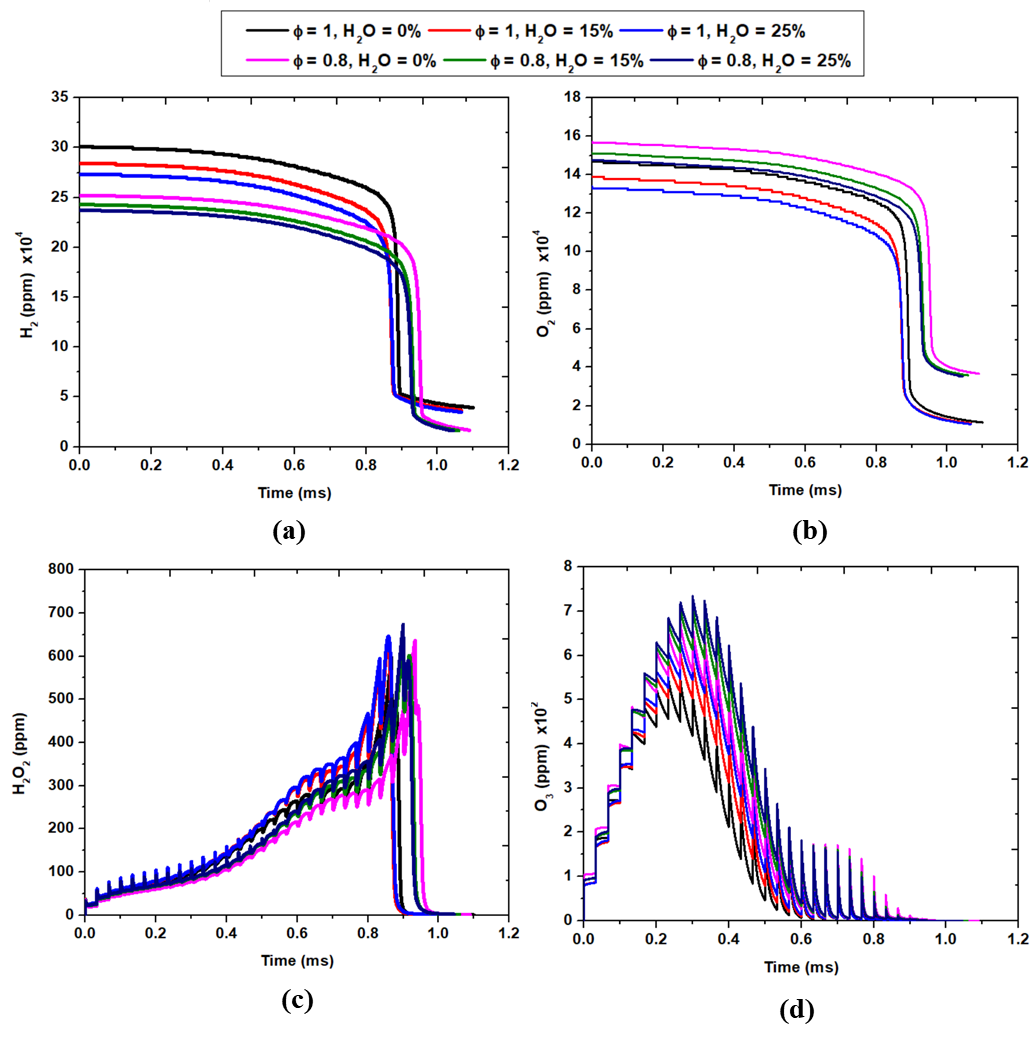

Supplement: Supplementary file 1 [file mmc1.zip › Research Data/Pure Hydrogen results.docx]
